# Monitoring systems for public health program and policy implementation in community settings: features and suggested actions from a scoping review

**DOI:** 10.1186/s13012-026-01491-6

**Published:** 2026-03-27

**Authors:** Alison Zucca, Cassandra Lane, William Pascoe, Belinda Peden, Carly Gardner, Adam Shoesmith, Angela Smith, Edward Riley-Gibson, Andrew Milat, Tanja Kuchenmüller, Davi Mamblona Marques Romao, Luke Wolfenden, Nicole Nathan

**Affiliations:** 1 College of Health, Medicine and Wellbeing, University of Newcastle, Newcastle, NSW 2318 Australia; 2 Hunter New England Population Health, Hunter New England Local Health District, Wallsend, NSW 2287 Australia; 3 Population Health Program, Hunter Medical Research Institute, New Lambton Heights, NSW Australia; 4 National Centre of Implementation Science, University of Newcastle, Callaghan, NSW 2308 Australia; 5 Hunter New England Health Libraries, Hunter New England Local Health District, New Lambton, NSW Australia; 6 School of Public Health, University of Sydney, Camperdown, NSW Australia; 7 Sydney Health Partners, Sydney, NSW Australia; 8 Science for Health Department, World Health Organization, Geneva, Switzerland

**Keywords:** Review, Public health, Implementation science, Program evaluation, Global health, Qualitative evidence synthesis, Best fit framework synthesis

## Abstract

**Background:**

Monitoring systems are important for evaluating key outcome measures, identifying opportunities for improvement, and informing public health investment. While disease surveillance systems are highly developed and widely used, monitoring systems for the implementation of public health programs and policies in community settings are less established. To address this gap, this review aimed to: 1) describe the scope of the literature on implementation monitoring systems and their operational features; and 2) synthesise this literature to produce features and suggested actions for system design.

**Methods:**

A systematic search of five databases and grey literature sources was conducted to identify systems, frameworks, or guidance for monitoring the implementation of public health programs or policies in community settings. Studies focused on clinical healthcare, or disease surveillance were excluded. Two authors independently screened titles, abstracts, and full texts for eligibility. Included documents were then categorised by ‘Case’ (one or more documents exploring the same monitoring system, framework, or topic). For Research Aim 1, characteristic data for each Case were extracted and narratively summarised. For Research Aim 2, Best Fit Framework Synthesis was employed using an a priori framework informed by disease surveillance models. Full text of included documents were coded, and the framework iteratively modified, to develop a new framework with key features and suggested actions relevant to implementation monitoring systems in community settings.

**Results:**

Ninety-seven documents were identified, describing 75 distinct real-world implementation monitoring Cases. *Aim 1:* Most Cases were intended for use in high-income countries (64%) and focused on monitoring programs (81%) rather than policy. The most common topic areas related to nutrition (36%) and reproductive, HIV, and sexual health (28%). Primary responsibility for monitoring systems was most often held by national-level agencies (43%). *Aim 2:* Synthesis led to 13 key features of monitoring systems, with corresponding suggested actions across five broad Action Areas: 1) planning and preparation; 2) data collection activities; 3) system appraisal; 4) partner engagement, and 5) system revision.

**Conclusions:**

Findings emphasise that monitoring systems require attention across multiple Action Areas, including planning and resourcing, data collection, partner engagement, and system improvements (through both proactive appraisals and real-time response). This manuscript offers a foundational framework to guide policymakers and practitioners in monitoring the implementation of community-based public health policy or programs.

**Supplementary Information:**

The online version contains supplementary material available at https://doi.org/10.1186/s13012-026-01491-6.

Contributions to the literature

- We identified 97 documents describing 75 distinct Cases of existing or historical monitoring systems, guidance or recommendations about monitoring systems, or discussions about monitoring as part of a broader system.
- The qualitative evidence synthesis provides a foundation that can guide policymakers and practitioners in establishing routine implementation monitoring systems in community settings.
- We identified 13 key features which were summarised into five broad action areas: (1) planning and preparation; 2) monitoring data collection activities; 3) system appraisal; 4) partner engagement, and 5) system revision.
- Corresponding suggested actions were produced which could be used to improve the design and functioning of monitoring systems.

## Background

Implementation monitoring systems involve “*the continuous and systematic collection, orderly consolidation of pertinent implementation data with prompt dissemination of results to those who need to know*” [1] (definition adapted from public health surveillance frameworks). Implementation monitoring systems can serve a range of important functions within health and community systems to improve the quality and impact of health and social services. For example, monitoring the implementation of health programs and policies can help identify divergences between actual and recommended practice and its variation across contexts and populations. In this way, implementation monitoring systems can identify evidence-practice gaps and inform targeted investments or adaptations to implementation strategies to address them. Implementation monitoring can also identify unanticipated problems with implementation processes, inform ongoing communication with stakeholders about implementation progress, success, and sustainment, and enhance accountability among funders, policymakers, and practitioners engaged in efforts to improve policy or program implementation [2–4]. 

Within implementation science, monitoring systems can be conceptualised as an implementation strategy – i.e., used to enhance the uptake and sustainment of programs or practices [5]. For example, the Expert Recommendations for Implementing Change (ERIC) taxonomy includes: *develop and organise quality monitoring systems*, *and develop and implement tools for quality monitoring* [6]. Monitoring systems can improve implementation through multiple mechanisms of action, including accountability (e.g., making performance visible, prompting reflection and self-regulation, social comparison via benchmarking) and advocacy (using data to persuade stakeholders, communicate program value, and secure ongoing support) [7]. They also generate actionable information to support learning: at the practitioner level, monitoring systems underpin audit and feedback strategies, while at the systems level, they enable performance improvement and adaption of other strategies [6, 8]. It is this ability to inform the adaption of other implementation strategies that positions monitoring systems as a meta-strategy. That is, in addition to directly influencing implementation outcomes, monitoring systems also operate in the causal pathway between companion implementation strategies and outcomes. By shaping how companion strategies are adjusted over time, monitoring systems influence their effectiveness**.** Accordingly, monitoring systems are recommended as important infrastructure of health systems [9].

Implementation monitoring systems vary in their scale, reflecting the geographical or jurisdictional level at which a program or policy is implemented, and the governance structures responsible for oversight. At the local level, monitoring systems have been used in efforts to introduce new models of care, or to align professional practice with clinical practice guideline recommendations within medical units, clinics or hospitals [10, 11]. For example, the Asthma Management and Outcomes Monitoring System (AMOMS) is used during consultations as a clinician-reminder tool to prompt and document best-practice asthma care, while at the same time capturing data that can be extracted for ongoing performance metrics at the clinic level [8, 12]. At broader organisational, regional, or national levels, routine monitoring and disclosure of health system activity and performance against evidence-based care standards are used for healthcare transparency and accountability [13–15]. Government agencies have also established monitoring systems to track, evaluate and inform ongoing improvements in the implementation of public health prevention programs in community settings, such as early childhood education and care centres [16] and schools [17, 18] or to track government commitments to international frameworks, such as the Framework Convention on Tobacco Control [19], and global action aligned with World Health Organization (WHO) strategies and other initiatives [20–22].

Implementation monitoring in clinical settings (e.g., hospitals, health clinics) differs contextually from community settings (e.g., early childhood education and care centres, educational facilities, sports clubs, workplaces or broad community). As such, the operational features of monitoring systems may also vary, suggesting value in examining them separately. In healthcare, medico-legal considerations may influence the type of implementation data collected and how it is disseminated and used [23]. Clinical settings typically have a rich data environment, where existing data infrastructure is embedded in service delivery, enabling the capture of implementation data as a natural by-product of routine care. For example, adherence to standards of care, such as wait times for surgery, is routinely captured via hospital administrative data bases and electronic health medical records [13]. While new standards of care may initially be monitored through separate audits or “add-ons”, many (e.g., adherence to sepsis bundle care), have since been fully integrated into electronic health records [24, 25].

In contrast, community-based health promotion programs and policies generally lack embedded data systems in the organisations they target, where healthcare is not the primary mission [26]. Consequently, community-based monitoring systems often need to be purpose built, and can be costly, face structural challenges (e.g., legal, ethical, and practical barriers) [27–29], and be difficult to maintain over the long term [3, 30]. For example, the Population Health Information Management System (PHIMS) in New South Wales Australia was developed to track school- and an early childhood-based health promotion program across the state, capturing data on reach (e.g., number and proportion of schools and early childhood services participating, number of children enrolled in participating schools or centres), fidelity (e.g., completion of program practices / implementation targets at the site-level), implementation support (e.g., number of visits to sites to provide support, type of support provided during visits) and adherence to policy targets at school, program, and regional levels (e.g., implementation fidelity status at the local health district-level) [31, 32]. Establishing PHIMS required designing a new system and extensive development time to integrate multiple data sources and support reliable, actionable monitoring in a context where routine data did not previously exist. Specifically, this involved developing new electronic data collection tools that could be used by Health Promotion Officers. Timely feedback was operationalised through a real-time data dashboard, complemented by more periodic formal reports that could then be provided to health or education end users and partners. Another example is the Pacific Monitoring Alliance for Non-Communicable Disease (NCD) Action (MANA), established to monitor policy and system-level action across Pacific Island Countries and Territories. The MANA monitoring team compiled and validated data from multiple countries, which was presented on a public dashboard to track progress across policies and legislations (e.g., adoption of 6 tobacco, 6 food, 4 alcohol and 1 physical activity policies and legislations), governance (e.g., existence of a functional NCD taskforce, existence of national NCD indicators and targets), and health system indicators (e.g., national guidelines for the diagnosis and management of NCDs, availability of essential NCD medicines, availability of smoking cessation support, restricted marketing of breast milk substitutes etc.). This provided actionable information to support accountability, identify gaps, and guide coordinated improvements regionally [33].

To our knowledge, there is currently no systematic review (scoping or otherwise) that collates and synthesises data relating to the features of non-clinical public health implementation monitoring systems. The lack of such evidence is of concern, given the importance of non-clinical programs and policies for public health improvement, and of monitoring systems to identify opportunities to improve their implementation. We sought to address this critical evidence gap to support the development and execution of such systems. Specifically, we conducted a scoping review and a qualitative evidence synthesis of non-clinical public health implementation monitoring systems with two aims: 1) to scope and narratively summarise the current literature; and 2) to identify features of monitoring systems to provide suggested actions for their design.

## Methods

### Protocol and registration

Methods followed Johanna Briggs Institute [34, 35] and Cochrane [36], guidance and findings were reported per PRISMA Extension for Scoping Reviews [36, 37]. The protocol was developed by a multidisciplinary team of Australian-based university researchers, implementation scientists, and health service experts, experts from the WHO, and an expert WHO Editorial Board overseeing development of guidance, to enhance the implementation, scale-up, and sustainment of evidence-informed health interventions. The team also has extensive experience in conducting systematic and scoping reviews and have practical experience of implementation monitoring systems (NN, LW). Public involvement was not feasible in the design, conduct, reporting, or dissemination of the research.

## Eligibility criteria

### Types of documents

Included documents discussed routine non-clinical monitoring systems for the implementation of public health programs or policies. Any type of publication was eligible: reviews, commentaries, data-based papers, case dissertations, abstracts, guidelines, frameworks, manuals, and tools. Documents could be published in scholarly journals, or in the grey literature but were limited to those published in English.

Included documents focused on monitoring public health programs and policies (e.g., targeting physical activity; nutrition; sexual, HIV and reproductive health; mental health [38, 39]) in non-clinical settings (e.g., early childhood education and care centres, educational facilities, sports clubs, workplaces or broad community [40]). As such, documents were excluded if the systems monitored implementation solely in clinical settings (such as hospitals), and/or monitored clinical interventions delivered in the community (e.g., mobile health clinic), and/or involved medical or pharmaceutical therapy (e.g., vaccinations, medical prescriptions). While acknowledging the following interventions can indirectly influence health outcomes, for the purpose of this review we have also excluded non-health programs and policies targeting environmental health and safety (e.g., WASH [41]), food safety and security; education (such as those targeting academic outcomes and attendance); crime and justice; housing; unemployment; and other social interventions not directly targeting health. We included monitoring systems operating at the local, subnational, national and international levels.

Included documents explicitly described implementation monitoring systems and their features. Routine monitoring systems were defined as *“the continuous and systematic collection, orderly consolidation of pertinent implementation data with prompt dissemination of results to those who need to know*" [1]. We did not restrict our search to discussions of only current or historical monitoring systems. Rather we included documents providing recommendations, frameworks, and guidance for developing and implementing monitoring systems. Commentaries or conceptual discussions about monitoring systems were also considered. Documents were also included even if monitoring systems were not the primary focus of the paper. For instance, studies describing an implementation plan or evaluation process that incorporated a monitoring system as a specific step were considered. Additionally, documents describing measures or indicators for use in a monitoring system were also considered.

Excluded documents included those describing surveillance systems (i.e., systems tracking community health to identify and address diseases and health threats), or evaluation research (often a one-off activity that unfolded subsequent to the delivery and implementation of a program). Documents that did not describe at least one feature of an implementation monitoring system (real or conceptualised) such as necessary structures and support, data collection, management, and analysis activities, and dissemination of results were also excluded.

### Information sources and search strategy

We developed a search strategy with the assistance of an experienced information specialist and academic librarian, drawing on terms used in foundation papers [26, 42, 43] and following search pilot testing.

We systematically searched five electronic databases: Medline (OVID); Embase (OVID), PsycINFO (OVID) CINAHL (Ebsco) and Web of Science (Clarivate)—from inception to December 2023. The strategy combined MeSH terms and keywords for public health and health promotion (e.g., “Public Health,” “Health Promotion,” “prevent*”), policy and programs (e.g., “Health Policy,” “Policy Making,” “program*”), implementation and sustainment (e.g., “Implementation Science,” “Diffusion of Innovation,” “sustain*,” “scale-up”), and monitoring and performance (e.g., “monitor*,” “performance,” “audit*,” “evaluation,” “indicator*”) (Appendix A) Also, grey literature was searched across WHO, Centres for Disease Control and Prevention, International Development Agencies (UNICEF, USAID, and United Nations), and government websites (Australia, Canada, United States, Netherlands). Supplementary searches were undertaken in Google and Google Scholar limited to first 100 results via an incognito browser (January 2024 to April 2024). An expert working group of five implementation scientists and listed coauthors, identified additional literature. The reference lists and embedded links of eligible articles were reviewed to identify any additional eligible articles.

### Selection of sources of evidence

Search results from academic databases were exported to Endnote (Clarivate) for deduplication, then uploaded to Covidence (Veritas Health Innovation Ltd) for screening. Two reviewers (either WP, ER, AZ, ASh, CG, CL, BP, NN) independently screened titles, abstracts, and full-texts against the inclusion/exclusion criteria. Conflicts were resolved via consensus, and if necessary, a third reviewer consulted. All articles are listed in Appendix C.

Grey literature websites were searched using the phrase “monitoring system”, reflecting the single term/phrase most likely to capture eligible documents within the websites of organisations searched. Potentially relevant links were reviewed in full, with a second reviewer (AZ, ASh, or NN) confirming decisions.

### Aim 1. Scope of the literature

We used excel and REDCap [38] to extract, categorise, and consolidate data [44]. A data extraction template was developed, tested, and refined. Two independent extractors, consisting of a senior and junior public health researcher (AZ, WP), then used this template, cross-checking for accuracy. Qualitative coders cross-checked each data extraction, with any discrepancies discussed and resolved through team consensus.

### Data items

#### Document characteristics

We extracted four data items per document: year of publication, country of origin (determined by lead institution or author affiliation); and document type. Document types were categorised as follows:

1. For peer-reviewed academic publications identified from academic journals:
  ◦ Review: Systematic, scoping, or narrative literature reviews.
  ◦ Data-based publication: Quantitative, qualitative, or mixed methods.
  ◦ Non-data-based publication: Opinion or conceptual articles without primary data.
  ◦ Protocol: Planned or ongoing research descriptions.
  ◦ Conference abstract.
  ◦ Dissertation: Academic theses.
2. For non-academic publications:
  ◦ Data-based report: Publications by government or non-governmental organisations (NGOs) with primary or secondary data.
  ◦ Guideline/framework/manual/tools: Structured guidance, methodologies, or resources.
  ◦ Webpage: Online content outside formal reports.
  ◦ Other: Publications not fitting above (e.g., unpublished discussion papers).

#### Case characteristics

Next, we extracted data on the characteristics of monitoring systems across groups of documents that discussed the same content (hereafter referred to as a ‘Case’). A Case was defined as one or more documents focused on the same monitoring system, monitoring framework, monitoring guidance, or a conceptual discussion about monitoring. For instance, if the same monitoring system was described across three documents, these documents were grouped together as a single Case. Conversely, if only one document described a specific monitoring system, framework, or guidance, that document was treated as an individual Case.

Cases were charted using six data items:

1. Country income classification: economic condition of countries where the monitoring system features were described as applicable, using 2024 World Bank classification [40] High-, Upper Middle-, Lower Middle-, and/or Low income. We coded income classifications based on statements from the authors, and specific case studies and examples discussed within the document.
2. Responsibility level: Central agency(s) responsible for the strategic and/or practical oversight of the monitoring system ranging from international (highest level), to national, regional (e.g., provincial, state, municipality, prefecture), and local levels. For decentralised systems where strategic and practical responsibilities were distributed across agencies, the highest level or agency with the broadest geographic coverage was recorded.
3. Policy or program monitoring: whether a health program (single, multi-site) and/or health policy or guideline monitoring was described.
4. Policy or program target: disease, condition or health behaviour/s the program or policy sought to address.
5. Policy or program setting: settings in which the program or policy was being implemented, or where guidance applied.
6. Types of evidence in documents: the types of evidence provided across Case documents, including: review of literature; original data-based research, further classified as quantitative or qualitative studies; use of expert consultation; pilot testing; and author-generated statements, further categorised by level of supporting evidence (a) substantial use of literature and references, (b) experiential knowledge or case studies, or (c) limited use of references or examples.

Frequency counts and percentages describe the key characteristics of the monitoring literature using extracted variables.

### Aim 2. Synthesis of monitoring system features

To address Aim 2, we conducted Best Fit Framework Synthesis [45, 46], chosen using the RETREAT criteria for selecting a qualitative evidence synthesis approach [47]. We followed the stepwise process described by Booth and Carroll [48], led by a proficient qualitative researcher (CL). Table 1 provides full details for each step. Within Best Fit Framework Synthesis, an a priori framework derived from relevant models or theories serves as a scaffold to organise and structure the evidence [49, 50]. It prompts analysts to critically consider where changes are needed as they examine nuances and commonalities across the data, making iterative changes until the framework reflects the evidence under review. We built our a priori framework (Appendix B) drawing on established frameworks for disease and public health surveillance systems (Steps 2 and 3). As the primary purpose of monitoring and surveillance is aligned (e.g., timely identification and tracking, understanding health-related problems etc.) this provided a strong foundation to ascertain a new framework with features responsive to monitoring systems for public health program and policy implementation in community settings. To produce suggested actions, coders captured relevant content both through evidence-based findings, explicit and implied recommendations in the source documents, and through interpretive judgements about what appeared essential or consequential. The final set of features and corresponding suggested actions was informed by constant comparison, analytic notes, routine inter-coder discussion, and team expertise and consensus (Steps 4–7; Table 1).

**Table 1:** Application of Best Fit Framework Synthesis in the current study

RECOMMENDED STEPS [45]		CURRENT STUDY
Step 1	Synthesis aim	Aim was defined in the protocol: to identify features of public health implementation monitoring systems and to provide suggested actions for their design
Step 2	Identify relevant ‘best fit’ frameworks, conceptual models or theories	Relevant candidate documents and theories were identified; specifically, prominent guidance and best practice in disease and public health surveillance (a well-established field) [1, 3, 45–48]
Step 3	Generate the a priori framework	Following Step 2, the review team created a customised ‘meta-framework’ by integrating domains and concepts across the identified documents. The WHO guidance on communicable disease surveillance and response systems [1] formed a key contribution. A copy of the a priori framework can be found in Appendix B
Step 4	Code evidence from included studies against the a priori framework	Documents were grouped by ‘Case’. Three researchers (CL, BP, CG) undertook coding in QSR NVivo software, performing full-text reviews and assigning relevant text or screenshots (parts of figures) to framework categories. Coders first independently coded four documents to check inter-coder consistency [46]. Remaining documents were coded by a single coder
Step 5	Create new themes by performing secondary thematic analysis or thematic synthesis on any evidence that cannot be coded against the framework	Coders maintained analytic notes and met routinely to discuss emerging themes. The framework was iteratively refined to reflect emergent concepts and to accommodate evidence that did not clearly fit within the a priori structure
Steps 6 & 7	Produce new framework and revisit evidence to explore relationships between themes or concepts	The coders produced a new framework (revised with features and suggestions) representing public health implementation monitoring systems. This included a visual model and key concepts (broader variables and information on how features interacted) that had been identified in the process. The preliminary findings were presented to the broader team comprised of implementation science researchers and experts from the WHO, with complementary expertise in public health, monitoring systems, and evidence synthesis. It was then further developed via discourse, drawing on co-authors’ expertise but grounded in the findings. The team reviewed changes from the a priori framework, assessing dissonance and agreeance; discussed whether there were any observable variations by Case characteristic (e.g., low- and middle- income country vs high income country and policy vs program implementation); and agreed on key features and suggested actions within revised action areas [44] Coders represented their coded datasets, ensuring contributions across all Cases (emphasis on representation of Cases of diverse characteristics) The final output was a framework representative of the included Cases, detailing features of monitoring systems and corresponding suggested actions supported by the evidence

### Role of the funding source

This study was supported by funding from an NHMRC Centre for Research Excellence (APP1153479). The funder of the study had no role in study design, data collection, data analysis, data interpretation, or writing of the report.

## Results

### Search results

**Fig. 1:**
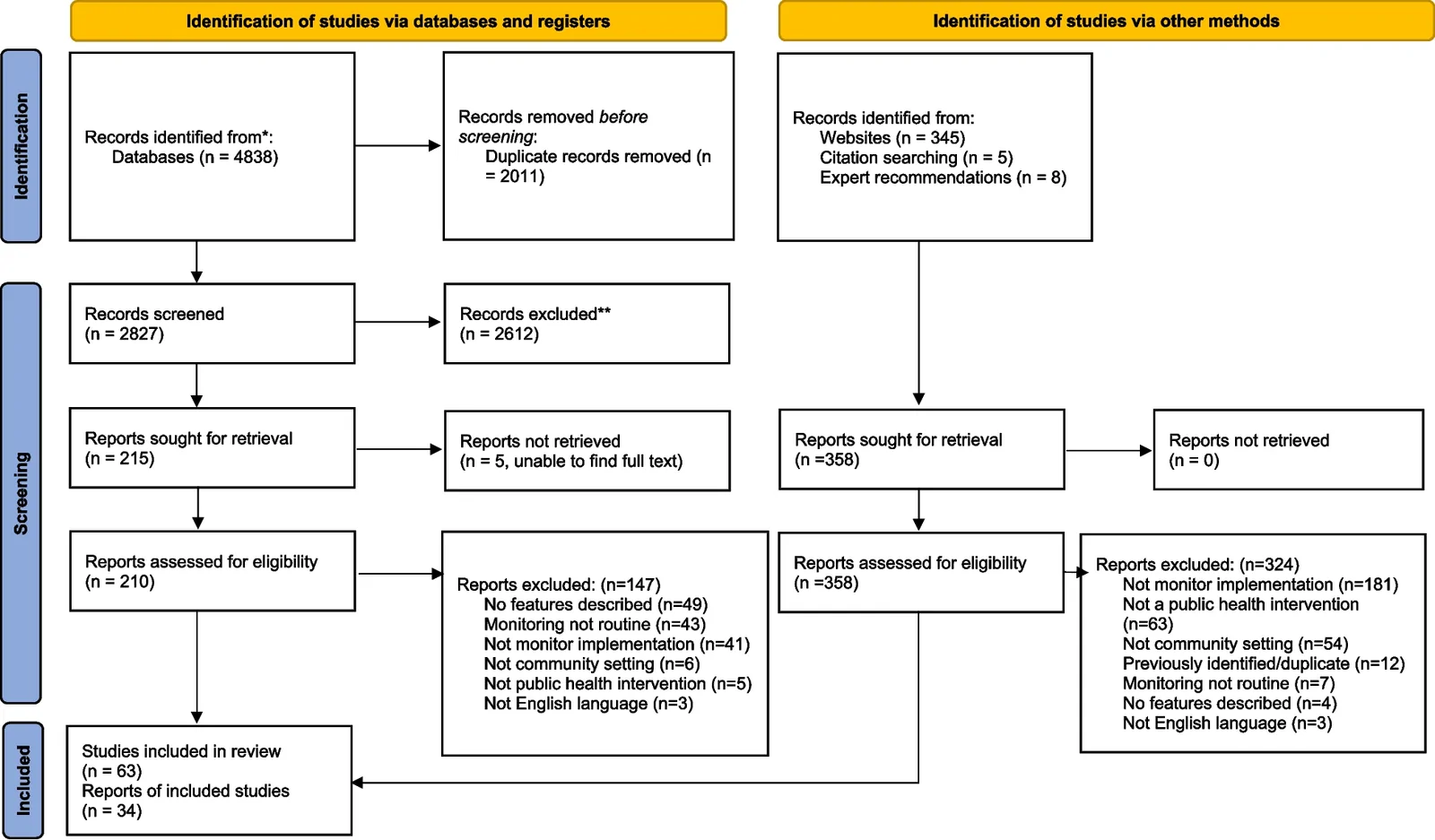
Scoping review PRISMA flow diagram

The academic literature search yielded 4838 results. After removal of duplicates, 2827 unique publications remained and were screened for eligibility, with 210 progressing to full text screening. An additional 358 records were identified through grey literature and forward citation searching. After full-text screening, 97 documents were deemed eligible and included for review (see Fig. 1; Appendix C).

### Aim 1. Scope of the literature

#### Document characteristics

Of the 97 documents, the majority were published in the previous ten years (2015–2024; 62%) and had primary authors affiliated with organisations based in a high-income country (94%), of which the most common was the United States (47%). The documents included a range of publication types, predominately non-databased peer-reviewed publications such as commentaries, positions papers, discussion pieces, and cases study descriptions (38%); followed by quantitative databased publications (18%), and frameworks, guidelines, tools published in the grey literature (13%). Reviews, identified as the most robust evidence source type, made up 5% of the total documents reviewed (Table 2).

**Table 2:** Document characteristics (*N* = 97)

		**N**	**(%)**
**Year of publication**	2015–2024	60	(62)
	2005–2014	31	(32)
	1995–2004	3	(3)
	1985–1994	3	(3)
**Document type**	Commentary	37	(38)
	Data-base quantitative	17	(18)
	Government/NGO frameworks, guidelines, tools	13	(13)
	Data-base qualitative	8	(8)
	Review	5	(5)
	Government, NGO data-based report	5	(5)
	Data-based mixed methods	5	(5)
	Dissertation	2	(2)
	Protocol paper	2	(2)
	Webpage	1	(1)
	Conference abstract	1	(1)
	Preliminary discussion paper	1	(1)
**Country of origin**^**a**^	**High income country**	**91**	**(94)**
	United States of America	46	(47)
	Australia	14	(14)
	Switzerland	10	(10)
	New Zealand	5	(5)
	Canada	5	(5)
	France	2	(2)
	United Kingdom	2	(2)
	Croatia	1	(1)
	Denmark	1	(1)
	Finland	1	(1)
	Ireland	1	(1)
	Japan	1	(1)
	Netherlands	1	(1)
	Portugal	1	(1)
	**Middle income**	**6**	**(6)**
	India	2	(2)
	Fiji	2	(2)
	Barbados	1	(1)
	Botswana	1	(1)
**Peer reviewed**	Yes	76	(78)
	No/Don’t know	21	(22)

^a^determined by the lead institution or author affiliation

#### Case characteristics

Eligible documents were grouped into 75 Cases. Overall, most Cases were based on a single document (*n* = 67, 89%), 5% (*n* = 4) on two documents, one Case on six documents, and another on ten documents. Cases predominately discussed monitoring in relation to high-income countries (48, 64%) and lower-middle income countries (44, 59%). Forty percent of cases (*n* = 30) were relevant to high-income countries only; 36% (*n* = 27) to middle- and/or low-income countries only; 19% (*n* = 14) were globally relevant across high-, middle- and low- income countries; and the remaining 5% (*n* = 4) were relevant to high-and middle-income only. Most focused on program implementation monitoring only (61, 81%), with nearly half involving monitoring of policy (33, 44%). The health target of these programs and policies was most commonly nutritional outcomes (36%), then reproductive, HIV and sexual health outcomes (28%). Cases discussed monitoring system settings most commonly within broad community settings (67%), followed by educational settings (25%). The highest level of agencies responsible for strategic and/or practical oversight of monitoring systems were primarily at the national level (32, 43%), followed by subnational (15, 20%) and international (13%) levels. The types of evidence across Case documents were most commonly generated by the author/s, primarily drawing from experiential knowledge and case studies (58, 77%). Original data-based research studies and literature reviews were across 16% of Cases, respectively (Table 3).

**Table 3:** Case* characteristics (*N* = 75)

Item	Variables	n	(%)
**Country income classification**^**a**^	High	48	(64)
	Upper Middle	33	(44)
	Lower Middle	44	(59)
	Low	30	(40)
**Responsibility level *highest agency-level responsible for managing the monitoring system***	International	10	(13)
	National	32	(43)
	Subnational (State/Region/Province)	15	(20)
	Local	7	(9)
	Single-site	2	(3)
	Unclear	9	(12)
**Policy or Program monitoring**^**a**^	Program only	42	(56)
	Policy only	14	(19)
	Both program and policy	19	(25)
**Policy or program target**^**a**^***health behaviour, condition or disease the program or policy sought to address***	Nutrition	27	(36)
	Reproductive, HIV and sexual health	21	(28)
	General/ not specified	18	(24)
	Alcohol, tobacco and other drugs	15	(20)
	Physical activity	13	(17)
	Mental health	6	(8)
	Malaria	1	(1)
**Policy or program setting **^**a**^	Broad community and/or households	50	(67)
	Educational (early childhood, schools, trade school or university)	19	(25)
	Community groups, centres and site (youth, peer-to-peer parenting, community distribution sites)	7	(9)
	Sports club	3	(4)
	Workplace	3	(4)
	Food retailers (restaurants, corner stores, vending machines)	2	(3)
	General or not specified	15	(20)
**Types of evidence in documents**^**a**^	Author generated	60	(80)
	*limited use of references or examples*	*2*	*(3)*
	*included experiential knowledge, case studies*	*47*	*(63)*
	*included substantial use of literature, references*	*32*	*(43)*
	Expert consultation	30	(41)
	Pilot testing mentioned (no data presented)	3	(4)
	Original data-based research	12	(16)
	*quantitative study*	*9*	*(12)*
	*qualitative study*	*7*	*(10)*
	Review of literature	12	(16)

^a^Total adds to more than 100% as multiple options were applicable

^*^ Case = one or more documents that explored the same content such as the same monitoring system, monitoring framework, monitoring guidance, or a conceptual discussion about monitoring. 89% (*n* = 67) of Cases were based on a single document, 5% (*n* = 4) on two documents, one Case on six documents, and another on ten documents

Forty percent of cases (*n* = 30) were relevant to high-income countries only; 36% (*n* = 27) to middle- and/or low-income countries only; 19% (*n* = 14) were globally relevant across high-, middle- and low- income countries; and the remaining 5% (*n* = 4) were relevant to high-and middle-income only.

### Aim 2. Synthesis of monitoring system features

Synthesis resulted in five broad action areas of monitoring systems, encompassing 13 features with corresponding suggested actions. A brief description of these action areas and features are described below and a detailed overview of each is provided in Table 4. Collectively, these action areas and suggestions describe the System—a hypothetical, proposed monitoring system based on this synthesis that represents optimal monitoring of public health program or policy implementation in community settings.

**Table 4:** Detailed results of data synthesis: action areas and features of monitoring systems with corresponding suggested actions

Action Area 1: Planning and preparing		
Feature	Overview of findings	Suggested actions
Purpose or objective *What the System intends to achieve*	The primary objective of each system typically reflected the needs of the Central Agency, with additional objectives often included to meet the needs of partners and end-users at more localised levels (including the implementation context). System objectives fell into the following broad categories: • Support scale-up: Systems aimed to facilitate policy or program evaluation across a greater number of areas/regions (vertical scale-up) and/or across different settings (horizontal scale-up). Notably, for several cases, monitoring was integrated within a broader scale-up process • Assess sustainment: Systems sought to track the impact, progress, equity, or implementation of a policy or program overtime, providing insight into sustainment • Inform decision making: Results were used as an advocacy tool to acquire support (e.g., funding) and to advise policy and action. They provided evidence to inform strategic decision making at relevant system levels • Hold agencies accountable: Results were used to hold responsible parties accountable for program or policy implementation, adherence, expenditures, or equity. They also served as benchmarks for comparing performance against comparable counterparts • Improve the policy or program: Results were used to identify opportunities for improvement (e.g., enhanced efficiency, impact, or equity, including addressing gaps such as missed settings or populations), which could then be addressed through targeted efforts	• Clearly define the purpose/objective(s) of the System as this will guide decision making • Consider including multiple objectives, e.g., how the System will meet the needs of partners or decision makers at each of the system levels. This will enable greater impact once the System is operationalised
Policy landscape *Influence of external policies*	The influence of external policies was a significant factor that shaped Systems across various contexts. In some Cases, the policy landscape acted as a driving force for agencies to undertake monitoring, for example, when objectives centred on measuring adherence to government mandates or directives. In other Cases, existing policies were leveraged to support monitoring activities or cited as essential drivers for initiating them. The policies referenced across Cases spanned three categories: • Direct policies: Policies with a specific focus on monitoring, explicitly designed to initiate or guide monitoring activities • Indirect policies—program or policy-related: Policies related to the program or policy being implemented, providing broader contextual support or guidance for the System without directly addressing monitoring • Indirect policies -health-related: Policies related to the health behaviour/s targeted by the monitored program or policy. For example, physical activity or healthy eating guidelines indirectly influenced monitoring activities	• Identify and prepare for any policies or external factors that may influence (facilitate or hinder) implementation of the System • Develop supportive policy mechanisms where none exist, including establishing a new policy or embedding monitoring requirements within an existing policy
Action plan *A blueprint for implementation*	Cases often referred to the importance of a resource detailing ‘how’ the System would be enacted (i.e., implementation guidance). The format and approach varied across Cases and included formal written documents; visual representations such as logic models; and existing theoretical or conceptual frameworks developed for the proposed system. Action plans typically covered multiple system features (*Action Areas 1–5*), outlining details such as roles and responsibilities, governance, data processes, ethical considerations, and decision pathways. They were often recommended to be co-developed with system partners (*Action Area 4: Engage*) and to remain flexible, allowing modification based on findings from system appraisal (*Action Area 3*) and ongoing system revision (*Action Area 5*)	• Develop a detailed action plan to serve as a blueprint for System implementation and a protocol for monitoring activities • Align the action plan with the cyclical logic of the System, ensuring it encompasses all action areas and features, including clear descriptions of how information will flow through the System (from data collection to data dissemination), and defined roles, responsibilities, and expectations of partners and implementers • Revise the action plan as needed (*Action Areas 3 and 5*)
Needs assessment *What is required for implementation*	Cases emphasised the importance of adequate resourcing to undertake monitoring activities (*Action Area 2*). As such, many included a needs assessment to identify required resources, generally related to: infrastructure (e.g., internet access in some locations), human resources (e.g., supervision, subject matter experts, workforce, training and development), financial resources (e.g., funding to support both income and expenses), and logistical supports (e.g., decision-support tools, defined time frames for feedback loops, and mechanisms to formally integrate monitoring system synthesis into decision-making, governance, technical assistance, and impact pathways). Partner identification and analysis was also described as important, used to confirm or ascertain interests of different groups and determine how to leverage support while managing potential risks (*Action Area 4: Engage*)	• Identify the resources and personnel required to implement and support monitoring activities, as specified in the action plan • Ensure the needs assessment encompass all system levels, from the Central Agency through to the implementation context, noting that this becomes more challenging as the System spans additional levels, due to increased number of needs and partners outside the Central Agency
Resourcing and capacity building *System supports*	The resources and infrastructure used to support systems varied depending on the context in which they were implemented. Cases highlighted several broad categories of support: • Infrastructure: Physical structures for operating the System, such as e-monitoring platforms (use of digital software or systems) and centralised technical support • Educational resources: Materials used to educat partners involved in monitoring activities • Teaching/training: Capacity building for staff engaged in monitoring activities (e.g., in-person workshops for data collectors) • Leadership and accountability: Structures to ensure effective leadership and oversight within the System • Partnerships and interest holder engagement: Efforts to engage identified interest holders and key partners in the monitoring process (*Action Area 4: Partner engagement*) • Organisational culture: Aspects of organisational culture that support monitoring activities • Communication and coordination: Mechanisms to facilitate effective communication and coordination among partners involved in monitoring Across Cases, these supports were described as foundational to collecting quality data (*Action Area 2: Data collection, Data quality*). Infrastructure such as capability and training of data collectors, and strong coordination across sites, directly shaped the validity, reliability, and comparability of data produced. Adequate resourcing was also established for other steps in the cycle, like system appraisal (*Action Area 3*) and ongoing partner engagement (*Action Area 4*)	• Based on the needs assessment results, allocate existing resources strategically to support the System, leveraging infrastructure or processes already used for program or policy implementation where possible • Establish additional resources and undertake capacity building activities to address outstanding needs, ensuring the System can perform across system levels • Prioritise the implementation context when allocating resources and building capacity
Action Area 2: Monitoring data collection activities		
Feature	Overview of findings	Recommendations
Indicators *What we need to know*	The indicators recommended or used by Cases aligned with Proctor et al.’s [49] three categories of implementation outcomes: Implementation outcomes (e.g., acceptability, adoption, reach, fidelity, feasibility etc.); Service outcomes (e.g., efficiency, equity, timeliness, safety); and Client outcomes (e.g., satisfaction, function, symptomology). Additionally, some systems aimed to measure mechanisms of implementation (e.g., contextual factors, barriers, and delivery dimensions). Some systems added or changed indicators over time as capacity (e.g., skills, data collection technologies) and resources increased (*Action Area 3: System appraisal; Action Area 5: System revision*) Cases also recommended selecting only indicators that were feasible within available System resources (*Action Area 2: Needs assessment; Resourcing)*, and prioritising equity by capturing differences within and between groups, with particular attention to priority populations	• Ensure indicators are clearly defined and aligned with the System objectives *(Action Area 1: Planning and preparing).* Consult existing literature to guide the selection of indicators appropriate to specific health topics or contexts
Data collection *The ‘how’*	Methods used to collect data for the indicators varied greatly depending on the System objective *(Action Area 1: Purpose or objective)* and whether monitoring was undertaken at the same level as the Central Agency or decentralised across more local levels. Across Cases, the most detailed reporting related to data collection, encompassing the components described below. • Type of data: Quantitative data were primarily used to measure indicator/s, likely due to ease of standardisation and amalgamation necessary for monitoring programs and policies (see *Data quality* below). Some Cases also incorporated qualitative data such as interviews, focus groups, document reviews, and observations. Data could represent either direct measures or proxy measures of the indicator • Data quality: Across Cases, decisions about who collected data, which sources were used, and the timing and frequency of collection were intended to support high-quality data. Quality data were most often described as valid (a true and accurate measure) and reliable (consistent and dependable across applications or over time). Cases also emphasised the importance of producing comparable data (e.g., via a standardised approach), enabling meaningful comparison and/or synthesis across sites and regions (*Action Area 2: Data synthesis*). This posed a greater challenge for decentralised systems, where maintaining external comparability could be difficult alongside data collection processes that are optimised for local relevance. Additionally, Cases recommended adequate coverage across data collection sites and regions, ethical data processes, and ensuring findings were interpretable for partners and decision makers (*Action Area 2: Data dissemination*). System appraisal (*Action Area 3*) and revision (*Action Area 5*) were described as important mechanisms for maintaining and strengthening data quality • Data collector/s: The person or entity responsible for collecting data originated from any of the system levels based on resourcing and who was best positioned to collect data for each indicator. Decisions about who collected data - and their capability - influenced data quality, shaped by factors such as proximity to the implementation context, training, and procedural consistency. Data collectors were internal or external to the Central Agency and could be directly involved with the program or policy, such as a program end-user (e.g., a survey completed by participant in a program) or delivery provider/implementer (e.g., a public health practitioner delivering a program); or a person or entity recruited specifically for data collection (e.g., outsourcing the responsibility to consultants or paid university researchers). • Internal data collector: An individual or entity from within the Agency • External data collector—program-specific: An individual or entity involved or associated with the policy or program being monitored • External data collector—outsider: An individual or entity recruited specifically for data collection •Source: Data were sourced through one of two means: (i) by accessing readily available data sources to measure the indicator(s) of interest, with relevant data having already been acquired, or being acquired, for other purposes; and/or (ii) by establishing a data source for monitoring purposes, either by incorporating data collection into an existing system or by creating a new data collection mechanism. Regardless of approach, across Cases decisions about data source were closely linked to data quality; for example, whether the source produced valid and reliable measures of the indicator or whether additional steps could be taken to establish validity. • Readily available data source: The indicator can be measured by sourcing data that is already existing, or in the process of being acquired, for other purposes • Establishing a data source: [adding on]. Sourcing data to measure the indicator by incorporating data collection methods within an existing procedure or data collection system • Establishing a data source: [entirely new], Sourcing data to measure the indicator by creating a new means of acquiring the required data •Timing and frequency: Timing of data collection was often specified in advance (*Action Area 1: Action Plan*) and suggested to occur “timely” or “routinely” in alignment with the system objective/s *(Action Area 1: Purpose or objective)* and dissemination needs *(Action Area 2: Data dissemination)*. Cases frequently described data collection occurring in real time (enabling rapid use), at routine intervals during program or policy implementation, or at the end of a program or policy cycle. *Note: Information regarding data management was limited. Few documents provided details on elements such as data storage; ownership/custodianship; privacy, security, and ethical considerations; or record-keeping. Where described, data management was often framed as part of planning (Action Area 1: Action Plan). Electronic and web-based data storage were the most commonly reported and were typically overseen by the Central Agency*	• Customise data collection methods for each indicator, selecting approaches with consideration to their acceptability, feasibility, and resource efficiency. Align methods with the capabilities and constraints of the System, and account for available resources, expertise, and infrastructure, and undertake capacity building where required. Data collection efforts are not limited to a single approach; use multiple actors, sources, and/or techniques where appropriate • A diverse array of methods (types of data, and perspectives) may assist in capturing a more comprehensive look at the outcome of interest; e.g., involve various partners to enrich understanding and triangulate findings by tapping into different perspectives and expertise • Prioritise data quality and ethics in data collection decisions, optimising validity, reliability, comparability across sites, and adequate coverage
Data synthesis *Amalgamating the collected data*	Data synthesis involved amalgamating collected data to derive information required for dissemination *(Action Area 2: Data dissemination).* Data were synthesised at different system levels to meet relevant system objective/s at each level *(Action Area 1: Purpose or objective)* *Note: Information regarding data synthesis was limited, with few documents describing details of these procedures. This made it difficult to synthesise or produce themes for the person responsible, the level and unit of analysis, and the methods used to analyse or synthesise data. In many Cases, synthesis was only briefly mentioned or inferred as part of broader data collection processes*	• Select synthesis methods with consideration to acceptability, feasibility, and resource efficiency. Align methods with the capabilities and constraints of the System, accounting for available resources, expertise, and infrastructure, and undertake capacity building where required • Make synthesis decisions collaboratively, engaging partners to ensure findings are relevant and actionable after dissemination
Data dissemination *Informing the target audience/s*	Data dissemination involved distributing the findings to the target audience/s to inform target audiences and/or action in alignment with the objective/s of the System *(Action Area 1: Purpose or objective).* Target audiences spanned all system levels, including Central Agency representatives, policymakers and other decision makers (more broadly), as well as program implementers, service providers, and the general public (more localised; within the implementation context). Various dissemination methods were employed, chosen with consideration to the most appropriate and suitable for each target audience. Across Cases, dissemination needs also shaped decisions about the timing of data collection (*Action Area 2: Data collection, Timing and frequency*), with data collected at intervals that enabled findings to be shared when most needed to inform action	• Disseminate findings to invested partners and decision makers in a timely and accessible manner to support the System objectives • Tailor communication approaches to the needs and preferences of different partner groups to maximise the impact and utility of findings • Explore additional opportunities for dissemination beyond the original System objectives (taking advantage of the knowledge); for example, sharing findings with the public to support advocacy or transparency, and publishing results to contribute to the evidence base
Action Area 3: System appraisal		
Feature	Overview of findings	Recommendations
Purpose *The reason for appraisal*	System appraisal processes were common among Cases. The purposes fell into three categories: (i) evaluating whether the System achieved its objectives; (ii) assessing System capacity, ensuring it remains on track and capable of meeting objectives; (iii) informing System improvements and facilitating continuous quality improvement	• Use system appraisal to assess progress toward System objectives and to inform continuous quality improvement
Approach *How the appraisal is conducted*	Responsibility for conducting System appraisal was typically held by the Central Agency, either conducted internally or outsourced to a partner, researcher, or other external entity. Methods varied and included both quantitative and qualitative approaches, ranging from ad hoc processes (e.g., feedback from partners or end-users) to formal research and evaluation activities. Appraisals were often conducted during a pilot phase and/or at the end of a complete cycle. In some Cases, assessments focused on a single action area or feature of the system (e.g., data collection or staff training), with appraisal occurring immediately after that activity. Appraisals of data quality (*Action Area 2: Data collection, Data quality*) examined validity, reliability, comparability, and ethics of the data, both to confirm quality and to identify challenges requiring refinement in data collection processes	• Plan system appraisal within the action plan, including distinct outcome measures and ways to ensure findings are used to improve the System • Conduct routine assessments of system performance, measured against predefined benchmarks to identify areas for improvement and implement corrective action
Action Area 4: Partner engagement		
Feature	Overview of findings	Recommendations
Engage *Engaging with partners*	Partner engagement was a cross-cutting component, consistently described as essential across all Action Areas. It was especially prominent in developing an action plan (i.e., co-production) and in resourcing and capacity building (*Action Area 1*), as well as in supporting effective and ethical data collection (*Action Area 2*). Engaging partners from the implementation context (those closest to where programs or policies were implemented and where data were collected) was especially important. Their perspectives informed indicator selection, supported feasible data collection methods (thus, increased likelihood of quality data [*Action Area 2: Data collection, Data quality*]), and enhanced interpretability of findings. Partners also contributed essential insights to system appraisal (*Action Area 3*) and revision (*Action Area 5*)	• Engage partners intentionally across the system cycle, ensuring they inform decisions and activities within each action area • Ensure representation of priority populations
Action Area 5: System revision		
Feature	Overview of findings	Recommendations
Revise *Responding to emergent needs*	System revision was another cross-cutting component, requiring responsiveness to emergent issues, needs, or opportunities for improvement across all action areas. This process was reactive, differing from the planned, proactive nature of system appraisal (*Action Area 3*). Some examples included seeking alternate resourcing (*Action Area 1*) or adjusting data collection methods (*Action Area 2*) to address in vivo barriers such as staff turnover. Like system appraisal, system revision played a key role in supporting data quality (*Action Area 2: Data collection, Data quality*), enabling real-time adjustments to ensure valid, reliable, and comparable data	• Remain attuned to emergent issues, needs, and opportunities, and make ongoing revisions to refine system operation
Key Terms and Concepts Case: One or more documents that explored the same content, such as an existing monitoring system, or a monitoring framework, monitoring guidance, or conceptual discussion about monitoring (related documents were grouped to be analysed as a single case) The System: The proposed monitoring system based on recommendations generated through this synthesis, representing optimal monitoring of the implementation of a public health policy or program in community settings System levels: The levels at which monitoring activities occur, including one or more of the following: international, national, state/region/province, and local Central Agency: The agency responsible for the strategic and/or practical oversight of the monitoring system (this may be situated at any of the system levels) Centralised approach: A model in which a single, central authority or organisation (e.g., a national health department) maintains ownership and control over the monitoring system Decentralised approach: A model in which the strategic and practical system responsibilities are distributed across agencies; this approach was reported to be more adaptable and responsive to local contexts Implementation context: The settings in which the public health program and policy is implemented and where monitoring data is collected		

**Fig. 2:**
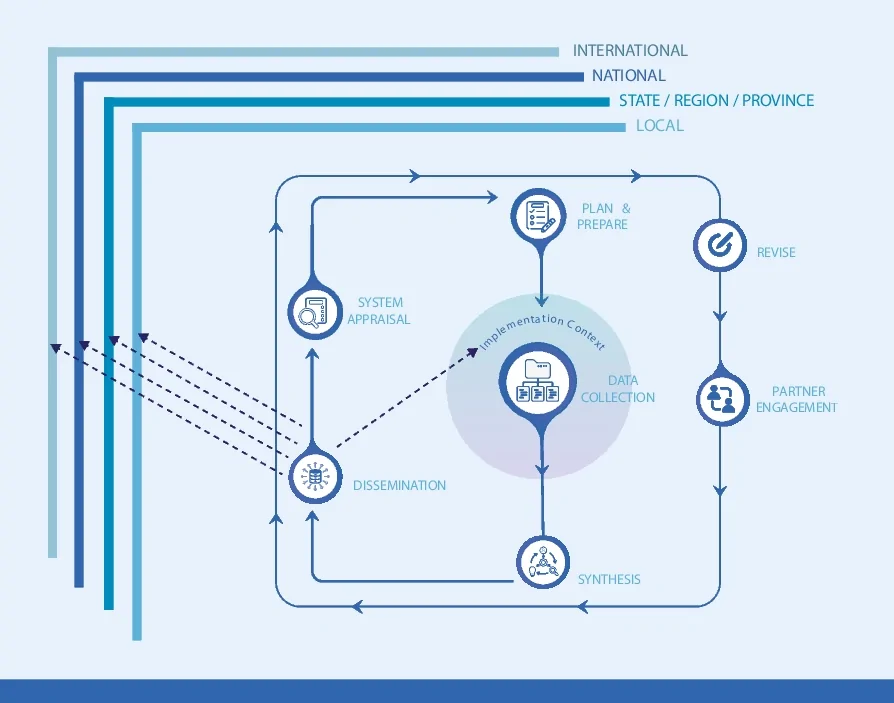
Diagrammatic representation of the System: a proposed monitoring system for public health policy or programs in community settings

Interpretation of the results is supported by several key concepts that emerged during synthesis. The System operates via a cyclical and iterative approach, centring on data collection within the implementation context—the settings in which public health program or policies are implemented. Monitoring activities cut across one or more system levels, including international, national, state/region/province, and local. The Central Agency, which holds strategic and/or practical oversight of the System, may operate at any of these levels and may do so using either a centralised approach (a single authority maintains ownership and control (e.g., [30, 51]) or a decentralised approach (strategic and practical responsibilities are distributed across agencies (e.g., [52, 53]). Figure 2 provides a conceptual representation of the System, showing how the action areas are situated across system levels and the implementation context at the centre. Appendix D presents three well-documented Case examples to exemplify features and suggestions.

#### Action Area 1: planning and preparing

This is a foundational step for the successful implementation of the System, ensuring the establishment of requisite structures and supports. This action area comprises five features: (i) forming monitoring objectives (e.g., to support scale up, assess sustainment, inform decision making, hold agencies accountable etc.) that capture the needs of the Central Agency as well as partners and end-users at more localised levels such as the implementation context; (ii) considering the policy landscape and how it might need to be addressed or leveraged to support the System or its activities; (iii) developing an action plan that provides guidance on how the system will be enacted; (iv) conducting a needs assessment to identify the resources required to undertaking monitoring activities (e.g., infrastructure, human, financial, and logistical); and (v), establishing resources and building capacity to prepare for monitoring activities and address identified gaps.

#### Action Area 2: monitoring data collection activities

The System relies on the systematic collection of high-quality data, followed by synthesis and dissemination to partners. The methods used or recommended in Cases varied depending on system context, approach (decentralised or centralised), and the nature of the monitored program or policy. The overarching goal of monitoring activities is to produce evidence that is sufficiently valid and reliable, and aligned with System objectives, to inform decision makers and partners. To enable meaningful interpretation after synthesis, many Cases used or recommended the use of standardised data collection and synthesis procedures that support data comparability.

The first feature of Action Area 2 is indicators, encompassing implementation outcomes (e.g., measures of quality or process care, policy or program delivery; acceptability; adoption; reach; fidelity; feasibility etc.), service outcomes (e.g., efficiency, equity, timeliness, safety), and client outcomes (e.g., satisfaction, function, symptomology), consistent with Proctor et al [5]. Indicators may also include mechanisms of implementation (e.g., contextual factors, barriers, and delivery dimensions). The second feature is data collection, which capture the methods used to measure indicators. This was the most extensively detailed feature across Cases and comprised several components: type of data, data quality, data collectors, data sources, and the timing and frequency of data collection.

For data type, quantitative data were most used, reflecting the need for standardisation for comparability across data collection sites or regions; and could be either direct or proxy data. Data quality was a central consideration – all data collection decisions were based on producing valid, reliable, and comparable data aligned with System objectives. Data collectors operated at various system levels and may be internal from within the Central Agency, external (associated with the policy or program being monitored), or recruited specifically for monitoring activities. Their proximity to the implementation context, training, and procedural consistency influenced data quality. For data source, it may be acquired from readily available sources (i.e., data already collected for other purposes) or by establishing a new source specifically for the System objectives. Finally, timing and frequency of data collection were typically specified in advance and were expected to occur routinely or in real time to support timely dissemination and decision-making.

The third feature is data synthesis, involving the amalgamation of data to produce information that responds to System objectives. Few Cases specified synthesis methods, limiting method-specific recommendations for the System. The final feature is data dissemination, involving tailored strategies to distribute the synthesis findings across system levels and inform action.

#### Action Area 3: system appraisal

System appraisal involves planned self-assessment at the end of a cycle to ensure the System is robust and achieves its objectives. In some Cases, appraisal involved evaluating the entire System while in others, it focused on specific features such as data collection or partner engagement. Some were even more focused, appraising discrete activities such as training for data collection personnel. Across approaches, appraising data collection methods to ensure high quality data was emphasised, possibly involving direct evaluation of the data itself. Following appraisal, the System may be enhanced, forming a cycle of continuous improvement.

#### Action Area 4: partner engagement

Central to the success of the System is the active engagement of partners at all system levels. This action area is an all-encompassing System feature rather than standalone, and is therefore depicted outside of the cycle in Fig. 2.

Partner engagement ensures relevance and alignment with priorities and needs of communities and decision makers. It facilitates buy-in, promotes transparency, fosters accountability, and enhances the utility of System findings. Engagement is also crucial for high-quality data, for example, through insights from data collectors and end-users that inform optimal data collection and interpretation. Multiple partnerships may present additional complexity due to the diverse needs and interests involved, with some partners posing potential risks to monitoring activities. Partner analysis can help identify these interests and outline ways to leverage support of some while managing risks posed by others.

#### Action Area 5: system revision

Across all broad action areas, the System requires ongoing, responsive revisions to refine its operation as issues and opportunities emerge. As another all-encompassing action area, it is also depicted outside of the cycle in Fig. 2. It allows the System to adjust in real time in response to emergent needs, challenges, and opportunities, e.g., those identified through informal stakeholder feedback or observations from System staff.

## Discussion

### Overview

This scoping review highlights the breadth of literature for monitoring systems of public health programs and policies in community settings, covering Cases from high-, middle- and low-income countries. Cases were predominately led by national or state agencies and explored implementation monitoring of program and/or policies targeting nutrition, reproductive and sexual health (including HIV), substance abuse, and physical activity.

Five broad action areas of monitoring systems were identified, comprising 13 features with corresponding suggestions. These features reflect the main tasks in designing and implementing monitoring systems, providing a foundational framework for actionable steps toward successful monitoring. Our findings reinforce the complexity of monitoring systems, characterised by diverse relationships between agencies and partners spanning from local to international levels, and adopting both centralised and decentralised approaches of ownership, management, and governance. Each of these approaches to monitoring warrants further exploration, including how to align system design with the aim and scope of monitoring. Here, we offer general guidance that is adaptable to various situations rather than precise instructions. This aligns with the Cases in this study, which showed nuanced differences required for successful monitoring, and reflects the field of public health’s recognition of the importance of context in the successful design and implementation of programs.

### Distinguishing implementation monitoring from surveillance systems

Our a priori framework was informed by key literature, including the WHO’s communicable disease surveillance and response systems framework [49] and STEPwise approach to noncommunicable disease risk factor surveillance [50]. This provided a useful foundation for refining and building the final framework. There were many similarities – particularly the emphasis on producing timely, complete, and useful data to inform action for public health benefit. It also enabled comparison between features of surveillance systems (a priori framework) and implementation monitoring (final framework).

The first notable distinction was partner engagement (Action Area 4). In the a priori framework, this feature was originally positioned under ‘Planning and preparing’. In contrast, during synthesis, partner engagement emerged as an overarching feature, to be integrated throughout all monitoring activities. This appeared to be related to the complexities and variability in monitoring public health programs and policies in community settings, which requires partners and end-users for responsive planning, determining indicators, data types and sources, and for undertaking or supporting data collection or other monitoring tasks. Accordingly, our final framework elevates partner engagement as a distinct action area. This aligns with broader implementation science literature in which meaningful, ongoing engagement of interest holders is recognised as foundational to successful implementation [54] and critical for supporting health equity [55]. Where implementation has been done well, the relationships, trust, and communication established during policy or program implementation can be leveraged to support monitoring. This is reflected, for example, in Learning Health System models, which engages interest holders throughout the implementation cycle, including monitoring activities [56–59].

Data quality (Action Area 2) was another notable distinction between the a priori framework and final framework. Communicable disease surveillance systems typically operate within mandatory disease reporting systems, where health care providers and/or laboratories report cases to a centralised database with established data quality standards [60, 61]. Our synthesis showed that monitoring public health programs and policies in community settings often require a more subjective and tailored approach to data collection. For example, in many Cases, data were gathered directly by service providers or end-users, introducing potential response and social desirability biases (e.g., [62, 63]). Several Cases emphasised the importance of appraising not only the system, but also the data to identify and address data quality risks [22, 64–67]. Planning and investing in quality data collection procedures, infrastructure, resources, and capacity building can support successful monitoring systems [64].

It is also important to note that some features in the a priori framework were poorly covered in the reviewed documents, including details on sampling methods, selection criteria (e.g., targeting all intervention settings or a specific subset), data management, and data synthesis. These gaps likely reflect the highly idiosyncratic and context-specific nature of monitoring systems, where such details are not considered generalisable or are deemed unworthy of reporting and are thus omitted. It is also possible that the level of detail on these aspects might exceed the scope of many documents included in this review. Regardless, this information is crucial to refine and improve features and recommendations provided in this paper. Further exploration with agencies managing monitoring systems may clarify these aspects.

### Governance across system levels

Findings from Aim 1 showed that responsibility for the strategic and practical oversight of monitoring systems were primarily at a high level (i.e., the national level). However, during the process of coding for Aim 2, we observed that oversight could be distributed across multiple levels. For example, decentralised monitoring systems were characterised by distributed ownership, control, and data collection responsibilities across agencies. This often involved using local methods within a shared overarching governance framework or guidance (e.g., [42, 68]). In contrast, centralised monitoring systems were overseen by a single Central Agency—such as a national department of health—which maintained ownership and control over the design, implementation, and management of the system (e.g.,[21]). Each model presents unique advantages and challenges that require consideration and may differ based on the scope of each system. For example, in lower-level or smaller-scale systems, such as those operating at the organisational, service, or local community level, monitoring activities are closer in proximity to the implementation context, and thus, may enhance responsiveness to emergent needs (Action Area 5) and facilitate closer engagement with key partners and interest holders (Action Area 4).

### Implications for health equity

It is important to note the potential for implementation monitoring to advance efforts to address health equity. Implementation is a critical point at which to address health inequities, ensuring that evidence-based programs and policies reach and benefit priority populations, typically defined as subgroups “*within a dimension of inequality such as age, gender, ethnicity, income or geographical location*” [69]. Implementation monitoring systems can support this goal by enabling the disaggregation of data to identify gaps for priority populations. This was reflected across many of the Cases in the current study, including guidance from the WHO which emphasised: *“It is… now crucially important to reinforce and reform national health information systems to ensure that they have the capacity to collect, analyse and report equity-relevant data, and to support the systematic integration and use of such data in decision-making”* [69, 70]. Implementation monitoring systems can also contribute by generating evidence, such as information on contextual factors (including equity-relevant determinants) that impede implementation, to inform remedial action [70–72]. In the proposed System, the inclusion of ongoing partner engagement as a core action area, consistent with participatory implementation science models [55], can support the ethical collection, interpretation, and use of equity-relevant data, particularly through leadership by, or co-production with, priority populations [55]. The WHO provides several resources that can support monitoring systems to incorporate equity considerations across the cycle [70].

### Methodological reflections

Most of the evidence included in the synthesis was author-generated content, often based on experiential knowledge or case studies, rather than from findings of more rigorous study designs that could empirically identify optimal features or assess their relative effectiveness. However, only two Cases with author-generated content lacked supporting examples or literature; the remainder were well supported through case studies, implementation experience, and/or extensive citation of prior evidence. Several parts of our analytic approach also enhance confidence in the synthesis findings. For example, the use of NVivo provided a transparent audit trail for coding and results development [73]; and involvement of the broader research team, including members with expertise in the topic area, helped to review concepts, prompt reflection, and finalise features and suggestions [46]. Data triangulation further enhanced study rigour. Our data set was large (75 Cases), diverse, and representative of monitoring systems implemented at multiple levels (local to international) and across country income classifications. This breadth and variation enabled systematic comparison across Cases, supporting assessments of agreement and dissonance, and development of a more complete understanding of features and corresponding suggested actions [74].

Taken together, these strategies provide a strong qualitative-derived foundation for the synthesis findings. However, consistent with qualitative evidence syntheses, the suggestions generated here are not intended to be rule-bound or prescriptive. As Hannes and Lockwood [75] emphasise, users “*…need to read and apply them in their given context and in conjunction with their own knowledge and experience as well as relevant consumer perspectives that are important in their particular local environment”.* Empirical research will be valuable to extend and refine the suggestions in the current study, distinguishing effective from ineffective monitoring system features and actions, and identifying which approaches are most appropriate for particular system contexts and goals, including under different governance arrangements, resource constraints, and system sizes.

### Limitations

We also acknowledge limitations of this study. First, some policy papers were excluded because they were one-off audits or mapping studies describing frameworks for benchmarking policies against international best practices rather than routine monitoring systems. Papers discussing the development of monitoring measures or indicators without mentioning a subsequent monitoring system with which to administer them were also excluded. Although these documents were not within the scope of this review, they may offer valuable insights for future studies. Second, we included documents in English- language-only (reflecting the official language of the WHO), which may have missed important contributions, particularly in the grey literature, from non-English language sources. Third, because our dataset included several scoping reviews, it is possible that some underlying primary studies were represented more than once across different documents. However, our synthesis was not frequency-based so any such duplication is unlikely to have influenced the conceptual findings. Fourth, we did not formally compare synthesis results based on specific Case characteristics (e.g., whether monitoring systems focused on programs or policies, were applied in low-, middle- or high-income countries, or operate at smaller versus larger system scales). This was discussed by the team in Steps 6 and 7 of the synthesis process (see Table 1), where the coders confirmed that they did not observe meaningful divergences in the features or suggested actions across these categories. Notably, the coders observed strong alignment between the overall findings and the coding for Cases from both low-income; and lower middle- income countries. This is likely due to the substantial contribution of Cases applicable in low-income countries (40%) and low- and middle-income countries (59%), many of which contained rich data across Action Areas and features. Future research is needed to formally examine whether and how implementation monitoring may operate differently across different contexts. Finally, our scoping review was restricted to public health monitoring systems in community settings, excluding those in clinical settings. While the synthesis findings may have relevance for clinical contexts, the organisational structures, workflows, and implementation supports characteristic of clinical settings could introduce key differences. Future research is needed to examine monitoring systems in clinical settings and to explore how their features compare with those identified in the current study.

## Conclusion

This scoping review consolidates and integrates information about monitoring systems for public health programs and policies in community settings. It provides a strong foundational framework with guidance for decision-makers interested in planning, operationalising, and/or improving monitoring systems. The features and suggested actions produced are robust and comprehensive—drawing on a synthesis of the best available published literature on monitoring systems across low-, middle- and high-income countries, a variety of health topics, and inclusive of existing systems and guidance documents. Researchers, policy makers, and health system leaders may use these findings to support the development of their own monitoring systems while also serving as a scaffold for further development and adaptation as more evidence emerges about monitoring systems of public health programs and policies. For instance, these findings could serve as the initial framework for future framework synthesis or as a basis for consensus-building activities with policymakers and practitioners to guide the next stages in the research agenda.

## Supplementary Information


Supplementary Material 1.Supplementary Material 2.

## Data Availability

This scoping review is based on analysis of data extracted from publicly available sources (published peer-reviewed literature and grey literature). All data generated or analysed during this study are included in this published article and its supplementary information files. Additional information can be made available upon reasonable request to the corresponding author.

## Abbreviations

HIV: Human Immunodeficiency Virus
HMRI: Hunter Medical Research Institute
MRFF: Medical Research Future Fund
NCOIS: National Centre of Implementation Science
NGO: Non-governmental Organisation
NHMRC: National Health and Medical Research Council
PRSIMA: Preferred Reporting Items for Systematic reviews and Meta-Analyses
PRSP: Prevention Research Support Program
*REDCap*: Research Electronic Data Capture
UNICEF: United Nations International Children's Emergency Fund
USAID: United States Agency for International Development
WHO: World Health Organization
