# Supplementary material for: Monitoring systems for public health program and policy implementation in community settings: features and suggested actions from a scoping review
Source: Implement Sci. 2026 Mar 27;21:49. doi: 10.1186/s13012-026-01491-6 (PMC13528058; doi:10.1186/s13012-026-01491-6)
Supplement: Supplementary file 2 [file 13012_2026_1491_MOESM1_ESM.zip › Appendix.docx]

**Appendix A: Search Strategy**

**Database: Ovid MEDLINE(R) and Epub Ahead of Print, In-Process, In-Data-Review & Other Non-Indexed Citations and Daily <1946 to December 15, 2023>**
**Search Strategy:**
**1**  Public Health/ (96836)
**2**  Public Health Practice/ (5682)
**3**  Health Promotion/ (82031)
**4**  1 or 2 or 3 (178102)
**5**  exp Health Policy/ (133856)
**6**  Policy Making/ (18032)
**7**  5 or 6 (145207)
**8**  4 and 7 (18283)
**9**  ((health or public or prevent*) adj2 (policy* or policies or program or programs* or programme or programmes)).ti,ab,kw,kf. (164506)
**10**  8 or 9 (178499)
**11**  Health Plan Implementation/ (6638)
**12**  "Diffusion of innovation"/ (18341)
**13**  Implementation science/ (1352)
**14**  ((implementation or engagement or sustainment or sustainability or scal*-up) adj3 (policy* or policies or program or programs* or programme or programmes)).ti,ab,kw,kf. (25469)
**15**  (implementation adj1 (strat* or adapt*)).ti,ab,kw,kf. (7663)
**16**  11 or 12 or 13 or 14 or 15 (57147)
**17**  10 and 16 (7517)
**18**  (monitor* or e-monitor* or performance).ti,ab,kw,kf. (2293314)
**19**  17 and 18 (1032)
**20**  limit 19 to english language (987)

**Database: Embase <1947 to present>**
**Search Strategy:**
**1**  public health/ (251391)
**2**  exp health promotion/ (118011)
**3**  1 or 2 (358470)
**4**  exp health care policy/ (227401)
**5**  3 and 4 (32896)
**6**  ((health or public or prevent*) adj2 (policy* or policies or program or programs* or programme or programmes)).ti,ab,kw,kf. (192136)
**7**  5 or 6 (215853)
**8**  implementation science/ (5227)
**9**  "diffusion of innovation"/ (173)
**10**  ((implementation or engagement or sustainment or sustainability or scal*-up) adj3 (policy* or policies or program or programs* or programme or programmes)).ti,ab,kw,kf. (32076)
**11**  (implementation adj1 (strat* or adapt*)).ti,ab,kw,kf. (8880)
**12**  8 or 9 or 10 or 11 (44740)
**13**  7 and 12 (7505)
**14**  (monitor* or e-monitor* or performance).ti,ab,kw,kf. (2934553)
**15**  13 and 14 (1127)
**16**  limit 15 to english language (1086)

**Database: APA PsycInfo <1806 to December Week 1 2023>**
**Search Strategy:**
**1**  exp public health/ (38078)
**2**  health promotion/ (29111)
**3**  1 or 2 (64055)
**4**  exp health care policy/ (14641)
**5**  policy making/ (31644)
**6**  government policy making/ (22638)
**7**  4 or 5 or 6 (65832)
**8**  3 and 7 (5229)
**9**  ((health or public or prevent*) adj2 (policy* or policies or program or programs* or programme or programmes)).ti,ab,id. (70983)
**10**  8 or 9 (73639)
**11**  implementation.ti,ab,id. (114746)
**12**  (monitor* or e-monitor* or performance).ti,ab,id. (530769)
**13**  10 and 11 and 12 (689)
**14**  limit 13 to english language (652)

**Web of Science**

(((health OR "public health" OR prevent* ) NEAR/2 (policy* OR policies OR program OR programs* OR programme OR programmes ))) AND ((((implementation OR engagement OR sustainment OR sustainability OR scal*-up ) NEAR/3 (policy* OR policies OR program OR programs* OR programme OR programmes ))) OR ((implementation NEAR/1 (strat* OR adapt* )))) AND ((monitor* OR e-monitor* OR performance )) Limit to English Results 1260

**CINHAL**

**Appendix B: Pre-determined codebook for A-priori code book**

| 1. **PLAN AND PREPARE** | | | |
| --- | --- | --- | --- |
| **MONITORING SYSTEM STRUCTURE** | | | |
| Legislation | Regulatory framework (i.e., policy or legislation in support of monitoring) | | |
| Objective(s) | What is the purpose, objective, and/or priority of monitoring (why the system?) -e.g., early warning, detect changes in practices, prioritize resource allocation… | | |
| Implementation level(s) | Level at which monitoring system is implemented (what level is it taking place):   - Macro (national/federal) - Meso (state/provincial/region) - Micro (program, institute, department) | | |
| Networking & partnerships | Concerted efforts and collaboration between stakeholders and partners | | |
| Exploration & assessment | Assessment(s) to identify gaps and recommendations for the system supports (e.g., environmental scanning, needs assessment; literature reviews) | | |
| **MONITORING SYSTEM SUPPORTS** | | | |
| Policies & procedures | Policies and procedures to support the system (e.g., an action plan, blueprint, protocol, description of roles and responsibilities, etc. – details of how the monitoring will work) | | |
| Resources | Resources used to operationalise the system:   - Financial: Internal or external funding - Human: People and/or partners - Logistical: Tangible resources | | |
| Training | Building capacity of staff involved (e.g., training manuals, modules, courses etc.) | | |
| Supervision | Strengthens and oversees system | | |
| Communication | Communication mechanisms (conveying of information) which support reporting and feedback in the system (including communication facilities, processes, tools, methods) | | |
| Coordination | Effective coordination between implementers and partners | | |
| Maintenance | Maintenance mechanisms: anything in place to support the ongoing maintenance. improvement of the monitoring system | | |
| 2. **CONDUCT MONITORING ACTIVITIES** | | |  |
| **DATA COLLECTION** | | |  |
| Type | What data is collected AKA the ‘indicators’ or ‘implementation measures’ | |  |
| Required elements | What is necessary to be collected; because not all information is necessary to support public health practice | |  |
| Level of collection | Level at which data is collected - what level it takes place   - Macro (national/federal) - Meso (state/provincial/region) - Micro (program, institute, department) | |  |
| Methods | How will data be collected? One approach or >1 approach? Quantitative and/or qualitative?   - Quantitative (e.g., surveys, case studies, observations, routinely collected data) - Qualitative (e.g., document review, focus groups, interviews, SWOT analysis) | |  |
| Duration | For how long data is collected | |  |
| Timing & frequency | How often data is collected | |  |
| Source(s) | Where data is acquired (e.g., health registers, test results, lab records, surveillance bulletins, case reports, previous monitoring data, online source) | |  |
| Person(s) responsible | Who is responsible for collecting the data (e.g., individual, group, organisation, department) | |  |
| Data quality | Elements representing quality such as completeness, simplicity, flexibility, sensitivity, acceptability | |  |
| Barriers/Limitations | Factors which may impede on data collection (and should be considered if not explicitly recommended) | |  |
| **DATA MANAGEMENT** | | |  |
| Storage | Where and how the data is stored | |  |
| Person(s) responsible | Who is responsible for overseeing data management (e.g., individual, group, organisation, department etc.) including data governance | |  |
| Ownership of data | Who owns the data | |  |
| Privacy, security, and ethical issues | Permissions to use the collected data | |  |
| Records | Audit trail, record-keeping | |  |
| **DATA ANALYSIS/SYNTHESIS/INTERPRETATION** | | |  |
| Person(s) responsible | Who is responsible for analysing the data (e.g., individual, group, organisation, department etc.) | |  |
| Level of analysis | Level at which data is analysed - what level it takes place   - Macro (national/federal) - Meso (state/provincial/region) - Micro (program, institute, department) | |  |
| Timing & frequency | How often will it be analysed | |  |
| Method of analysis | How data is analysed (e.g., quantitative, qualitative; manual or automated) | |  |
| Unit of analysis | The level at which data is assessed (prompt: will indicators reported separately by population and disaggregated by gender and age group where relevant and useful) | |  |
| Barriers/Limitations | Factors which may impede on data analysis (and should be considered if not explicitly recommended) | |  |
| 3. **FOLLOW-UP** | | | |
| **DISSEMINATION OF RESULTS** | | | |
| Person(s) responsible | Who disseminates the results | | |
| Method(s) | How results are disseminated | | |
| Target audience | To whom the results are disseminated – i.e., who will scrutinise the data? Which meetings/boards will this be reported to? | | |
| Level of dissemination | Level at which data is disseminated:   - Macro (national/federal) - Meso (state/provincial/region) - Micro (program, institute, department) | | |
| Timing & frequency | How often dissemination activities occur | | |
| Use & application | What happens after dissemination, what is the data used for? (e.g., advocacy, health promotion, changing/adapting implementation) | | |
| **SYSTEM APPRAISAL** *(this is focused on assessing the monitoring system/practices itself - any assessments of the prior elements in the codebook)* | | | |
| Use | The objective - why is appraisal of the monitoring system conducted; for what will this data be used for? | | |
| Quality assurance | Procedures, checks, audits, and/or corrective actions to ensure that all activities are of the highest achievable quality. | | |
| Implementation indicators | Acceptability, cost-effectiveness, flexibility, relevance, etc. [*of the monitoring system*] | | |
| System improvements | Informing improvements - a better way to do things, improve the system over time | | |
| Formal research | Formal evaluation/research/study undertaken to assess the monitoring system | | |
| 4. **OTHER** | | | |
| Recommendations | | Explicit recommendations made for the overall monitoring system; or interpreted as such due to consistent or strong referral by author(s) as an essential component/feature | |
| Limitations | | Factors which may hinder the overall system (perceived or actual) | |
| Benefits/Advantages | | Explicit benefits, advantages, and/or positive outcomes resulting from the monitoring system (perceived or actual) | |

**Appendix C: Unit of analysis and references [see attached]**

**Appendix D. An overview of three Case examples**

CASE EXAMPLE 1: [United States, National]: CDC’s Prevention Status Reports (PSRs)

The United States Centre for Disease Control (CDC; System Agency) Prevention Status Reports (PSRs) are used to monitor the status of evidence-based policies and practices targeting 10 key health concerns: alcohol; food safety; health care-associated infections; heart disease and stroke; HIV; motor vehicle injuries; nutrition, physical activity, and obesity; prescription drug overdose; teen pregnancy; and tobacco use. The CDC developed the system using a thorough process of planning and preparation in consultation with six state health departments, experts, and partners. This included review and feedback to proposed formats and materials, and an iterative process of prototype testing and refinement. The PSRs measure two indicators: implementation fidelity (using a 3-level scale of green, yellow, and red) and reach (comparing distribution of ratings over time) of policies and practices addressing the identified health concerns. Data are collected by public health professionals, community leaders, and policy makers within each state using a standardised, technical approach (supported through data entry into Excel data workbooks). Quality assurance checks are undertaken before results are shared, with data internally reviewed by CDC measurement experts and subject matter experts, and externally reviewed by state health departments and partners. In addition to being published online, the results are distributed across multiple levels of government. At the highest level, a national summary with aggregated information is produced to monitor performance and improvements for the indicators across the country. At the state-level, summaries and topic-specific reports for each state are used to inform decision making and foster dialogue with partners. The results may drive state health department efforts to improve low PSR ratings and serve as a resource to apply for federal funds. Finally, although local-level data are not included in the PSRs, local health departments may use the PSRs as a tool for localised assessment and the state-level data as a benchmark for comparison.

CASE EXAMPLE 2: Pacific Island Countries and Territories, Transnational]: Pacific Monitoring Alliance for Non-Communicable Disease (MANA) [45–47]

The Pacific Monitoring Alliance for Non-Communicable Disease (MANA) Dashboard is a transnational, collaborative platform that assesses Pacific region countries progress on implementing NCD prevention programs and related policies [46]. This system developed out of a policy landscape where all 21 Pacific nations endorsed a NCD Roadmap and a process to monitor progress of this plan was needed [47]. MANA aims to increase accountability on NCD action with a focus on building each county’s capacity, both in planning and prioritising NCD responses and in technical data capacity. During the early implementation of the MANA monitoring system, training was delivered to build the capacity of public health professionals (data collectors) to collect more accurate data through a course ‘Data for Decision Making’. This was rolled out across the region [45]. The 31 indicators MANA uses were based on those set out in the WHO NCD Progress Monitor and expanded to suit the region’s context and monitoring objectives. The dashboard reports and rates progress on these indicators in each country using a traffic light and star ranking system. The traffic light system indicates progress on the action/policy (red=none, amber= in development and green = in place) then a star system is used to demonstrate the strength of the action (zero – three) stars [47]. Data are collected in a variety of ways, including the team co-ordinating the MANA process using publicly available information to pre-fill the dashboard which is then verified and amended. Findings from the monitoring process and annual reports are disseminated at all meetings of the Pacific Heads of Health and Health Ministers and released as peer-reviewed publications (Baseline Data 2017-2018: [47] and follow up data: 2019 [46]).

CASE EXAMPLE 3: [National, multiple countries, particularly LMICs]: Flour fortification [17,48]. The *WHO Monitoring flour fortification to maximise health benefits: A manual for millers, regulators and programme managers* provides expert guidance for country-level monitoring of a cost-effective program that increases micronutrient content in flour to address malnutrition and nutrition-associated health outcomes. Flour fortification program partners reach across national-level agencies with responsibility for fortification as well as industry partners across the supply chain, research institutions and consumer-protection groups. Responsible agency objectives included ensuring the fortified product is safe, contains the required level of micronutrients and is being consumed optimally. This enables rapid corrections to overcome issues identified through monitoring. Partners and end-users’ objectives included clearly defining the interplay between the roles and activities of different partners involved in the system and the overall operation of the system and demonstrate accountability for resource expenditure. The manual recommends that a clear and comprehensive purpose (taking into account the context of the program) is an important aspect to guide the design of the monitoring system. The policy landscape in many flour fortification countries includes a Food Act or Law which provides a clear mandate for activities. Considerations for undertaking a needs assessment and developing an action plan are outlined in a process labelled as describing the programme which includes a needs assessment, expected outcomes, contextual factors, stages of development, resources needed and a logic model. Resourcing (e.g., financial, material and technical) for each activity within the monitoring system and capacity building (e.g., initial and refresher training) is recommended. In acknowledgement of the challenges within country-specific contexts, the manual recommends determining indicators based on feasibility to track and provide the best evidence of program performance. Data collection is undertaken at various levels using a number of methods, including household surveys, and it is recommended that existing sources of information are assessed first for utility and accuracy before creating new data collection processes using the logic of data that can be easily collected and efficiently synthesised and disseminated throughout the various levels of the monitoring system. Periodic appraisal of the monitoring system is recommended to improve process efficiency, utility and data quality.
