# Supplementary material for: Monitoring systems for public health program and policy implementation in community settings: features and suggested actions from a scoping review
Source: Implement Sci. 2026 Mar 27;21:49. doi: 10.1186/s13012-026-01491-6 (PMC13528058; doi:10.1186/s13012-026-01491-6)
Supplement: Supplementary file 2 [file 13012_2026_1491_MOESM1_ESM.zip › AppendixC_UNIT OF ANALYSIS_ESM.docx]

| **UNIT OF ANALYSIS/CASE** | **TYPE** | **Country Income**  (ie, High, Middle, Low) | **Highest level of responsible System Agency** | **Focus: Public health topic** | **Setting of policy or program** | **Level of evidence behind recommendations /features** | **Brief overview of the elements of the paper that are coded and relevant to this scoping review** | **Reference** |
| --- | --- | --- | --- | --- | --- | --- | --- | --- |
| **Population Health Information Management System (PHIMS)** | Monitoring system | High | State (NSW, Australia) | Program: Childhood obesity, physical activity, nutrition | Schools | Mixed methods descriptive case study, ethnographic qualitative study with users of the monitoring system, author/s generated ideas with substantial referencing, | Series of documents describing the development and evaluation of the PHIMS monitoring system | Conte et al. (2017)^1^ |
|  |  |  |  |  |  |  |  | Conte et al. (2019)^2^ |
|  |  |  |  |  |  |  |  | Conte et al. (2020)^3^ |
|  |  |  |  |  |  |  |  | Goldberg et al. (2022)^4^ |
|  |  |  |  |  |  |  |  | Green et al. (2018)^5^ |
|  |  |  |  |  |  |  |  | Bravo et al. (2020)^6^ |
|  |  |  |  |  |  |  |  | Loblay et al. (2022)^7^ |
|  |  |  |  |  |  |  |  | Gron et al. (2020)^8^ |
|  |  |  |  |  |  |  |  | Farrell et al. (2014)^9^ |
|  |  |  |  |  |  |  |  | Innes-Hughes et al. (2019)^10^ |
| **Necessary components for effective program implementation** | guidance /recommendations (indirect) | All | Not specified | Policy and Program: General | Broad community | Author generated with limited referencing and brief examples | Briefly discusses the features of measuring the performance and implementation of public health programs. | Frieden (2013)^11^ |
| **IMPACT implementation support platform** | Monitoring as one component | High | General /not specified (USA) | Program: (examples) Mental health, alcohol tobacco and other drugs, sexual health | Schools (example), Broad community (example) | Author generated using case studies and examples | Explores a generalised (rather than bespoke) software data system designed for monitoring the implementation of a variety of public health interventions. | Weaver & DeRosier (2019)^12^ |
| **Flour fortification** | Monitoring system | All | National | Program: Nutrition | Broad community | Guidance developed by several key international organisations based on technical knowledge and field experience, using published frameworks, examples, and substantial referencing. | Guidance for a monitoring system for a national-level flour fortification processes. | WHO (2021b)^13^ |
|  |  |  |  |  |  |  |  | Pena-Rosas et al. (2008)^14^ |
| **CDCs Prevention Status Reports (PSRs)** | Monitoring system | High | National (USA, 50 states) | Policy and Program: including Alcohol, Tobacco and other drugs; Sexual, Reproductive and HIV health, Nutrition, Physical activity, Food Safety, Motor Vehicle safety. | Various | PSR framework developed via stakeholder consultation (qualitative interviews), and iterative review. Indicators and rating scale developed via systematic review and search of guidelines, pilot testing, and expert panel. | Describes the design and development of a national public health performance measurement system. | Young et al. (2018)^15^ |
| **Communities That Care (CTC) Prevention System** | Monitoring as one component | High | Local (12 communities, across 7 states of USA) | Program: Adolescent drugs, delinquency | Schools; after-school programs, broad community, | Author generated using experiential knowledge and some referencing | Describes the development and application of monitoring component of the Communities That Care (CTC) prevention framework. | Fagan et al. (2008a)^16^ |
|  |  |  |  |  |  |  |  | Fagan et al. (2008b)^17^ |
| **Scaling up EBIs in US Public Systems to Prevent Behavioral Health Problems** | Other [expert consensus and commentary on scale up factors] | High | State (unspecified examples, USA) | Policy and Program: General | Various (example) | Author generated using examples and substantial referencing | Describes the various factors that affect EBI scale-up across public systems, one of which is data monitoring and evaluation capacity. | Fagan et al. (2019)^18^ |
| **Process evaluation of community-based behavioral intervention trial in Tanzania** | guidance /recommendations (direct) | Lower middle | Local (Dar es Salaam, Tanzania. USA research team responsible for overseeing) | Program: Sexual health, partner violence | Broad community | Author generated using case study example | Describes the 2-year process evaluation of an implementation intervention. | Balvanz et al. (2023)^19^ |
| **National Adolescent Health Programme (Rashtriya Kishore Swaasthya Karyakram or RKSK)** | Monitoring as one component | Lower middle | National (India, commissioned WHO review) | Program: Adolescent sexual and reproductive health | Community health centres, broad community | Rapid programme review conducted by the WHO to provide guidance to improve program implementation (including monitoring systems) | Provides recommendations for improving monitoring of the program. | Barua et al. (2020)^20^ |
| **HIV Prevention Programme Cascade** | Monitoring system | Lower middle | National (Kenya) | Program: HIV prevention for Female Sex Workers | Broad community | Author generated with substantial referencing of literature and using substantial experiential knowledge/case study/examples. | Describes the application of an HIV prevention programme cascade framework to monitor and improve HIV prevention interventions for female sex workers (FSWs) in Kenya. | Bhattacharjee et al. (2019)^21^ |
| **INFORMAS** | Monitoring system | High (examples) Upper middle Lower middle | National | Policy (audit and implementation): Nutrition, health food environments. | Broad community | Literature search of existing measures of policy implementation | Series of documents describing the development, evaluation and application of the INFORMAS monitoring system | Brinsden et al. (2013)^22^ |
|  |  |  | National (New Zealand) |  |  | Example of implementation monitoring in New Zealand |  | MacKay et al. (2022)^23^ |
|  |  |  | National |  |  | International experts developed policy monitoring process, author generated features using substantial references, |  | Swinburn et al. (2013a)^24^ |
|  |  |  | National (New Zealand) |  |  | Food-EPI pilot and psychometrics testing. |  | Vandevijvere, Mackay & Swinburn (2018)^25^ |
|  |  |  | National (Japan) |  |  | Example of implementation monitoring in Japan |  | Yamaguchi et al. (2022)^26^ |
|  |  |  | National |  |  | International experts developed policy monitoring process, author generated features using substantial references, |  | Swinburn et al (2013b)^27^ |
| **Federal level performance management (PM) of population health interventions federal policy** | guidance /recommendations (direct) | High | National (Canada) | Program: General | General /not specified | Author generated using substantial experiential knowledge/case study/examples. | Series of documents discussing applying performance measurement to federal public health programs | Boileau-Falardeau et al. (2021)^28^ |
|  |  |  |  |  |  | Author generated with substantial referencing of literature and using substantial experiential knowledge/case study/examples. |  | DeGroff et al. (2010)^29^ |
|  |  |  |  |  |  | Author generated with substantial referencing of literature, and using substantial experiential knowledge/case study/examples. |  | DeGroff (2010)^30^ |
| **Community-based youth PA program (VERB)** | Monitoring as one component | High | Local (Lexington Kentucky, USA) | Program: Physical activity | Broad community | Author generated with case study. | Briefly describes program tracking and evaluation used when delivering the VERB Summer Scorecard program. | Bryant et al. (2010)^31^ |
| **Evaluating National Malaria Control Programs in Africa** | guidance /recommendations (direct) | Upper middle Lower middle Low | National (African countries) | Program: Malaria | Broad community | Author generated with substantial referencing of literature. | Includes broad recommendations for national monitoring of Malaria control programme implementation in African countries | Bryce et al. (1994)^32^ |
| **Sweet potato Fortification in Kenya** | Monitoring as one component | Lower middle | Regional (West Kenya and involving national and international collaborators) | Program: Nutrition | Broad community. Maternal health clinic | Author generated with case study. | Discusses monitoring implementation of a multisectoral agricultural and health intervention | Cole et al. (2016)^33^ |
| **Monitoring Implementation of prevention programs (Guidance)** | commentary /special issue summary | High (examples) | General /not specified | Program: General/ non-specific | General /not specified | Author generated with substantial referencing of literature, and using substantial examples. | Discusses elements for inclusion in a data collection and reporting system, and provides an example of e-monitoring software. | DeRosier (2019)^34^ |
| **Mental health policy** | guidance /recommendations (direct) | All | National | Policy (audit and implementation): Mental health | General /not specified | WHO guidance includes international collaborators from various countries, with substantial referencing of literature. | Presents an approach for national-level monitoring of mental health policy. | Funk & Freeman (2011)^35^ |
|  | guidance /recommendations (indirect) | High | National (England) | Policy (audit): Mental health | General /not specified | Review and thematic synthesis of mental health policies in England. | Presents a thematic synthesis of mental health policies highlighting the importance of monitoring and accountability to reduce ethnic and racial inequalities in mental health services | Hussain et al. (2022)^36^ |
| **CDC standardized evaluation system for HIV prevention programs** | Monitoring as one component | High | National (USA, 65 health departments from USA states and territories including Puerto Ric, Virgin islands, America Samoa, Micronesia, Guam, Maria Islands, Marshall islands, Palau) | Program: HIV | General /not specified | Pilot testing of the guidance with 8 health departments and consultation with all health department Directors | Describes the CDC guidance that can be applied by health departments at the state level to evaluate HIV prevention programs | Glassman et al. (2002)^37^ |
| **Adolescent Health and school health programs in India** | Monitoring as one component | Lower middle | National (India) | Program: Adolescent reproductive and sexual health, hygiene; nutrition; alcohol tobacco and other drugs | Schools, Broad community | Rapid review of programs including monitoring. | Rapid review of the overall planning, implementation and monitoring of adolescent and school health programmes of India. | Jain et al. (2022)^38^ |
| **Public Health Policies (PA, nutrition, sedentary behaviour) in Europe** | Other | High Upper middle Lower middle | International (Policy Evaluation Network, 7 high income EU countries and NZ) | Policy (audit and implementation): Physical activity, nutrition | Broad community, Schools (examples) | Author generated with substantial referencing of literature, and using substantial examples. | Protocol paper describing the methods, including exploring monitoring progress in the implementation of a policy. | Lakerveld et al. (2020)^39^ |
| **Early Risers Prevention Program** | guidance /recommendations (indirect) | High | State (Minnesota, USA) | Program: Alcohol, Tobacco, and other drugs | Schools | Author generated with substantial referencing of literature, and using case study example. | Describes the feasibility of a technology designed to assess implementation fidelity of the Early Risers conduct problems prevention program across 27 geographically dispersed school sites. | Lee et al. (2008)^40^ |
| **Brazillian AIDs program** | guidance /recommendations (indirect) | Upper middle (example) Lower middle (guidance) Low (guidance) | International (e.g. world bank collaborating with Brazil) | Program and policies: Sexual, reproductive and HIV health | Broad community | Author generated with case study example. | Briefly mentions the importance of monitoring the organizational and political evolution of programmes to ensure sustainability of programs. | LeLoup et al. (2010)^41^ |
| **D.A.R.E Program** | guidance /recommendations (indirect) | High | State (50 states, USA) | Program: Other Drugs | Schools | Author generated with case study example. | Describes the D.A.R.E. national infrastructure for community-based prevention. The state-level D.A.R.E. entities are responsible for monitoring the quality of program implementation. | Merrill et al. (2006)^42^ |
| **School-based PA and nutrition program** | guidance /recommendations (indirect) | High | State or local (unclear) (USA) | Program: Physical activity; nutrition | Schools | Literature review | Literature review of approaches used to measure the implementation of school-based programs and policies. | McGraw et al. (2000)^43^ |
| **Sustainment Measurement System (SMS)- (U.S. Substance Abuse and Mental Health Services)** | Monitoring as one component | High | National (USA) | Program: Mental health; Substance abuse; Underage alcohol prevention | Various: High schools; community | Author generated with substantial referencing and use of implementation guidelines and frameworks. | Protocol for a study that will be used to develop a monitoring system for grantees delivering public health interventions. | Palinkas et al. (2016)^44^ |
| **University Nutrition Policy** | Monitoring as one component | High | Single site (University of California Berkeley, USA) | Organisational single-site policy: Nutrition | University | Author generated with case study example /substantial experiential knowledge. | Discusses a university’s experience with policy development, implementation, monitoring and evaluation. | Rickrode_ Fernandez et al. (2021)^45^ |
| **EBIs** | Other (commentary) | High | General /not specified (discusses USA) | Program: Mental Health | General /not specified | Author generated with limited referencing. | Briefly discusses embedding monitoring into mental health related EBIs. | Rotheram-Borus, Swendeman & Chorpita (2012)^46^ |
| **Monitoring progress on Health for All policy WHO** | Monitoring as one component | High | International (WHO) | Policy (audit of existing policies; implementation): General | General /not specified | Substantial referencing and broad use of case study examples in article. | Discusses monitoring of progress toward accepted international health policy goals. | Salmela (1991)^47^ |
| **Monitoring guidance: School Health Nutrition Programs** | guidance /recommendations (Direct) | All | National or state (>100 countries offering programs) | Program: Nutrition | Schools | Author generated drawn from case studies in many countries. | Discusses evidence and guidance for monitoring of school health and nutrition programs including: why monitor, what to monitor, how to monitor, and who monitors | Schultz & Ruel-Bergeron (2021)^48^ |
| **Technical Support Units (TSU) to support scale up** | Monitoring one component | Lower middle | State (Karnataka, India) | Program: Sexual and HIV health | Broad community, sexual health clinics | Description of one case study. | Discusses how a contracted Technical Support Unit facilitated monitoring. | Sgaier et al. (2014)^49^ |
| **CDC Program Evaluation and Monitoring System (PEMS)** | Monitoring system | High | National (USA) | Program: HIV | Broad community | Developed by Centre for Disease Control and Prevention (CDC), with stakeholder engagement at each stage of program - ongoing feedback; indicators developed via theory and literature reviews. | Discusses Program Evaluation and Monitoring System for HIV prevention and updates to improve the effectiveness and feasibility of this system. | Thomas, Smith & Wright-DeAgüero (2006)^50^ |
| **Community coalitions** | Monitoring system | High | State (rural QLD, Australia) | Program: cardiovascular disease, substance abuse, reproductive health | Broad community | Utilised previous research from CDC and WHO where possible. Describes one case study example. | Document the development and results of a web-based monitoring system for rural community health programs. | Turner et al. (2006)^51^ |
| **National health plans and strategies (NHS) monitoring** | guidance /recommendations (direct) | All | National | Program: General | Not specified | Expert opinion, author generated with substantial referencing, substantial experiential knowledge. | WHO provides guidance for a country-led monitoring of national health strategies. | WHO (2011)^52^ |
| **Global Strategy Monitoring (Infant & Child Feeding)** | Monitoring system | High Upper middle Lower middle | International (WHO/UNICEF, 18 European countries) | Policy (implementation); Program: Infant and young child nutrition, breast feeding | Broad community | Author generated with substantial experiential knowledge and case study. | Reports on monitoring system results including inter-country comparisons. | Zakarija-Grkovic et al. (2020)^53^ |
| **Health Service monitoring LMICs (Essential Packages of Health Services)** | Guidance/ Recommendations (direct) | Low & middle | National (Ethiopia and Pakistan, examples) | Policy and Program: general (cardiovascular disease, cancer) | Broad community; but largely clinical/primary health care delivery | Collaborative expert opinion, literature review, and case studies to inform guidance /recommendations. | Guidance for low- and middle-income countries in developing monitoring and evaluation of national health policy using detailed examples and expert opinions. | Danforth et al. (2023)^54^ |
| **Reach and Implementation (R&I) system for public health funded grants** | Monitoring system | High | State (Colorado, USA) | Program: General | Various: Not specified | Pilot testing to develop items and refine systems operation. Refined system implemented in 1 state across 2 years with 103 public health programs.  Included collaboration between policymakers and academics. | A standardised approach for collecting, analysing, and reporting data from all funded department of health projects from one state. | Fernald et al. (2012)^55^ |
| **Mobile for Reproductive Health (m4RH) SMS health information service** | Monitoring as one component | Low & middle | National (Tanzania, Rwanda, Uganda, Kenya) | Program: Reproductive health and family planning | Broad community | Author generated with substantial experiential knowledge and case study. | Describes an mHealth monitoring and evaluation system where user interaction data is continuously collected, and then used to inform intervention adaptations. | L'Engle, Plourde & Zan (2017)^56^ |
| **Uttar Pradesh Health and Management Information System (UP-HMIS)** | Monitoring system | Lower middle | State (Uttar Pradesh, India) | Policy: General health | Broad community; clinical health services | Qualitative interviews with system users; quantitative data exploring data quality; substantial experiential knowledge and case study. | Describes a monitoring system that captures data absent in Health Management Information systems and combines data from various government portals and health programs. | Meghani (2021)^57^ |
| **Health system strengthening** | Guidance/ Recommendations (direct) | Low & middle | General /not specified | Program: General community related health | All health services and systems: interrelated health facilities composed of health centres and hospitals, outreach services, community groups, and household. | Review of 15 health system strengthening documents identified via a systematic review. | Summarises the role of community in monitoring. | Sacks et al. (2017)^58^ |
| **Guidance on Process Evaluations** | Guidance/ Recommendations (direct) | High | Single program (USA) (Hypothetical) | Program: General, Alcohol & Tobacco (Hypothetical) | Schools, broad community, | Author generated guidance, drawing from the literature. Hypothetical case study used to illustrate guidance. | Describes the steps involved in developing a process evaluation plan for any health promotion program. | Saunders, Evans & Joshi (2005)^59^ |
| **Indicators for monitoring harm reduction service** | Other [consensus study] | High | International (~ 30 European countries) | Program: People who use drugs | Community needle and syringe program (also discusses monitoring implementation of medical therapy OAT) | Expert consensus to develop indicators and review existing quality standards. | Describes consensus on validated and internationally agreed upon best practice indicators for monitoring harm reduction services. | Wiessing et al. (2017)^60^ |
| **Communicable disease monitoring & surveillance** | guidance /recommendations (direct) | All | National /subnational: (194 WHO member states) | Program: General (Surveillance and response systems for communicable diseases) | Broad community | Expert opinion, author generated with substantial referencing, substantial experiential knowledge. | Describes a framework and list of indicators proposed for purposes of monitoring and evaluating surveillance and response systems to communicable diseases. | WHO (2004)^61^ |
| **Breastmilk substitution marketing monitoring** | guidance /recommendations (direct) | All | National | Policy (regulation): Nutrition and Breastfeeding | Various: Broad community, health services. | Developed by expert group (WHO), expert panel, substantial experiential knowledge from individual country case studies. | WHO toolkit to monitor the marketing of breast milk substitutes. | WHO (2017c)^62^ |
| **International Health Regulations** | guidance /recommendations (direct) | All | National (Countries who ratified IHR treaty own monitoring system, WHO provides leadership) | Policy (regulation): General health regulations | NA | Substantial experiential knowledge from individual country case studies. WHO expert leadership. | Monitoring compliance with IHR requirements - tools to support countries in monitoring their progress towards implementation of the IHR. | IHR (2005)^63^ |
| **Digital Health Intervention monitoring and evaluation** | guidance /recommendations (direct) | All | General /not specified | Program: General | Broad community | International expert panel of researchers and implementers, experiential knowledge and extensive use of literature and including frameworks. | Monitoring and evaluation guidelines for digital or mHealth interventions. | WHO (2016a)^64^ |
| **Women, Children & Adolescent Health Monitoring Framework** | guidance /recommendations (direct) | All | National | Policy and program: Sexual, reproductive and HIV health | Various: Broad community; clinical settings, | Literature review to identify existing indictors, stakeholder’s prioritisation exercise, open consultation process. | Discusses the development of indictors to monitor implementation and track progress towards women and adolescent sustainable development goals. | WHO (2016b)^65^ |
| **Home Fortification Interventions** | Monitoring system | Middle & low | Various levels: e.g. National, State and Local | Program: Nutrition | Broad community /household | Developed by US Centres for Disease Control and international collaborating experts drawing from frameworks, academic literature, and experiential knowledge. | Discusses how to monitor implementation of home fortification interventions. | WHO (2013)^66^ |
| **CDC School Health Policies and Practices Study (SHPPS)** | Monitoring system | High | National (USA) | Program and policy: physical activity, nutrition, mental health, | Schools | Survey items developed and revised via expert panel, cognitive testing /interviews with 42 people. | CDC methods and results of the implementation of the whole school child health policy in US schools. | CDC (2016)^67^ |
| **CDC online resource: 'Monitor and Evaluate' nutrition programs** | guidance /recommendations (indirect) | High | Regional (USA) | Policy and program: Nutrition | Community facilities /canteens and vending machines, including those in the broad community /public facilities, workplaces, sports clubs, health care facilities, | Developed by CDC monitoring tool content experts with reference to research, and via experiential knowledge and case studies. | Brief summary of key factors in monitoring food initiatives with links to CDC monitoring tool. | CDC (2024)^68^ |
| **National level food fortification** | Monitoring system | All | National | Policy and program: Nutrition | Broad community /household | Guidance from various experts via a global summit, advisory group, working group, technical group. | National food fortification monitoring recommendations. | UNICEF (2018a)^69^ |
|  |  |  |  |  |  | Case studies referenced from various sources | Implementation monitoring case study examples of national food fortification programs. | UNICEF (2018b)^70^ |
| **USAID guidance on health systems strengthening (HSS)** | guidance /recommendations [direct] | Low & middle | National | Policy and Program: Health systems strengthening with the primary goal of sexual, reproductive and HIV health (reducing maternal and child mortality, and HIV/AIDS mortality) | Various: including health systems - clinical and broad community | Literature review of health systems strengthening interventions, literature review of indicators, use of literature and policy guidance documents, international expert panel. | Explores strengthening health systems and includes guidance on monitoring, evaluation, and learning. | Aqil et al. (2017)^71^ |
| **USAID Program Cycle Operational Policy** | guidance /recommendations [indirect] | Low & middle | National (for USA use in foreign countries) | Policy and program: various including sexual, reproductive and HIV health (reducing maternal and child mortality, and HIV/AIDS mortality) | Various: including broad community, health services. | Internal expertise at USAID, experiential knowledge, literature review including use of international guidelines. | Describes that USAID Program Cycle Operational Policy which outlines the framework and guidelines for how the United States Agency for International Development (USAID) designs, implements, monitors, and evaluates its development assistance programs. | USAID (2023)^72^ |
| **NSW Ministry of Health Guide to scaling up population health interventions** | guidance /recommendations (indirect) | High | National (Australia) | Program: General | General, not specified | Author generated with experiential knowledge. | A step-by-step guide for scaling up population health interventions. A few paragraphs discussing monitoring. | NSW MoH (2023)^73^ |
| **Emonitoring** | Other [one component within monitoring] | All | General, not specified | Program: General | General, not specified | Author generated substantial referencing and use of case studies. | A discussion piece on Emonitoring and its potential for public health. | Conte & Hawe (2018)^74^ |
| **Community Education Monitoring System (CEMS)** | Monitoring system | High | Regional (USA) | Program: CVD risk reduction (include physical activity, nutrition, tobacco) | Broad community | Author generated with substantial experiential knowledge and case study. | Community education monitoring system using data from multiple sources. | Flora et al. (1993)^75^ |
| **District Monitoring and Evaluation (M&E) Officer** | Other [one component within monitoring] | Upper middle (example) Lower middle Low | National (Botswana) | Program: General | Broad community | Qualitative interviews with M&E Officers. | Description of monitoring and evaluation program and experience of new staff in Botswana. | Mpofu et al. (2014)^76^ |
| **Caribbean Community (CARICOM) accountability instrument** | Monitoring system | Upper middle Lower middle | International (20 countries, mostly Caribbean, report to CARICOM) | Policy and program: Smoking, nutrition, physical activity | Various: broad community, workplace, schools | Implementation indicators derived by authors from Declaration of Port of Spain and revised with help from one expert. | Describes a grid used by CARICOM (Caribbean Community) to compare each country's achievements in NCD commitments (country-specific; national-level). | Samuels, Kirton & Guebert (2014)^77^ |
| **CDC funded State Public Health Actions** | guidance /recommendations (indirect) | High | National (USA) | Policy and Program: General | Various: incl. broad community, schools | To develop the performance measures, experts reviewed the purpose and intended outcome of each strategy in the logic model, use of validated measures where possible. | Overview of the monitoring of performance of a national level policy implemented by states. | Vaughan et al. (2017)^78^ |
| **Pakistan Early Child Development Scale-Up (PEDS)** | guidance /recommendations (indirect) | Lower middle | Local (Naushahro Feroze district, Sindh province, Pakistan) | Program: Nutrition, enrichment /stimulation | Broad community: Home visits, routine services, parenting groups | Author generated drawing from some literature and experiential knowledge. | Implementation evaluation and description of methods of maternal and child health program in Pakistan using female health workers in community settings. | Yousafzai, Rasheed & Siyal (2018)^79^ |
| **UN National HIV monitoring and evaluation (M&E) system** | guidance /recommendations (direct) | High  Upper middle (pilot tested here) Lower middle (pilot tested here) Low | National | Policy: HIV/AIDs | Broad community | Guidelines developed by expert international panel, and pilot tested in three countries. | Guidelines include monitoring the progress of implementing a national HIV monitoring and evaluation system. | UNAIDs (2009) ^80^ |
| **NSW Schools Physical Activity and Nutrition Survey (SPANS)** | Monitoring system | High | State (NSW, Australia) | Policy: Nutrition and physical activity | Schools | Author generated using literature to develop monitoring items. | Databased report of student physical activity and nutrition status. Two questions assessed physical activity policy implementation in schools. | Hardy et al. (2010)^81^ |
| **Focus Resources on Effective School Health (FRESH)** | guidance /recommendations (direct) | Low Lower middle Upper middle | National | Policy and Program: Nutrition, physical activity, HIV, sexual and reproductive, substance use | Schools | Developed by expert international organisations and technical experts, and substantial reviewing of literature. | Detailed description of monitoring implementation indicators and the recommended process of data collection.  No referencing. | UNESCO (2014)^82^ |
| **Monitor, Protect Offer Warn Enforce Raise (MPOWER) (Tobacco)** | Monitoring as one component of broader system | All | International (two thirds of all countries) | Policy and Program implementation: Tobacco | Broad community | Monitoring system developed by expert international organisations and technical experts, referencing the literature, provides case studies and data- based exploration into the acceptability and feasibility of the system. | An example of implementation monitoring with monitoring data presented. | WHO (2017)^83^ |
| **HEPA in the EU** | Monitoring as one component of broader system | High  Upper middle Lower middle | International (~28 European Union countries, aided by WHO Regional Office Europe) | Policy: physical activity | Broad community | Expert panel developed monitoring framework. Example of monitoring and data presented. | Describes HEPA monitoring framework to support policy making in the EU (with 23 indicators). An example with monitoring data is presented. | Whiting et al. (2021)^84^ |
| **Pacific Monitoring Alliance for NCD Action (MANA) Dashboard** | Monitoring system | Upper middle Lower middle Low | International (21 Pacific Island Countries and Territories, aided by Pacific Islands monitoring alliance for NCD) | Policy and programs targeting NCDs: Nutrition; Alcohol & Tobacco | Various | Monitoring system developed via literature review, international expert panel and technical partners such as WHO and CDC, and descriptions/case study of the monitoring systems being used and adapting features of these to fit this context. | Describes a monitoring system, and compares national data describing implementation progress over time. | WinTin et al. (2022)^85^ |
|  |  |  |  |  |  |  | Describes the development of monitoring system and provides case studies/examples. | Tolley et al. (2016)^86^ |
|  |  |  |  |  |  |  | Reports baseline MANA monitoring data for all 21 countries. | WinTin et al. (2020)^87^ |
| **Monitoring for health systems integration at the national level (patient centred approach)** | guidance /recommendations (direct) | (examples from) Lower middle Low | National (Zambia, Kenya, Ethiopia case studies) | Policy: Health services integration, including community based (examples HIV, TB, antenatal) | Integrated health services at the health service and individual community level. | Author generated using substantial referencing and examples. | Outlines an approach for monitoring integrated health services. States a theory-driven logic model is necessary in the health information system. | Reynolds &Sutherland (2013)^88^ |
| **Workshop and Road Map for Monitoring and Implementing HIV-related Policy Reforms** | Other [one feature of a monitoring system] | Upper middle Lower middle Low | International (Africa, Caribbean and Central America regions) | Policy: HIV | Various (implied) but not specified | International experts developed policy monitoring roadmap; Quantitative survey of workshop participants. | Describes Road Map for Monitoring and Implementing HIV Policy Reforms, and documents participants perspectives on HIV/AIDS policy monitoring and capacity building workshop. | Lane et al. (2016)^89^ |
| **Community-based monitoring system for HIV prevention in India** | Monitoring as one component | Lower middle | State (Andhra Pradesh, India) | Program: Sexual and HIV health | Broad community: STI clinics, community outreach, and field work | Ethnographic study exploring monitoring system includes observational and key informant interviews. | Ethnographic study (qual) exploring community-based monitoring systems designed for Female Sex Workers FSWs re - HIV prevention in India. | Biradavolu et al. (2014)^90^ |
| **Monitoring Nutrition Programs in LMICs** | guidance /recommendations (direct) | Upper middle Lower middle Low | District (8 developing nations, examples) | Program: child Nutrition | Broad community | Discussion paper include a review of 11 case studies (not per reviewed). | Series of recommendations based on monitoring nutrition programs in LMICs. | Levinson & Madzorera (2006)^91^ |
| **Right foods at the Right Time (Malawi)** | Monitoring system | Low | District (Ntchisi, Malawi) | Program: Infant and young child nutrition | Various: health centres, community distribution sites, households | 1 case study. Monitoring guided by national and international organisations /experts (world food program, world vision). | Describes lessons learned from the program’s monitoring system, and use of data for quick-response program improvement within the program's logical framework. | Bergeron et al. (2019)^92^ |
| **The Physical Activity Environment Policy Index (PA-EPI)** | Monitoring system | High (example) | National (Ireland) | Policy: Physical activity | Various: Education, workplaces, sports clubs, and broad community - transport, urban design | PA-EPI monitoring framework developed by literature review, expert panel, quantitative and qualitative research. | Data-based paper explores the extent of policy implementation in the Republic of Ireland using the PA-EPI tool that identifies strengths and weaknesses to address during implementation. | Volf et al. (2023)^93^ |
| **Guidance for monitoring infant and young child feeding (IYCF) programs** | Guidance /recommendation (direct) | Upper middle Lower middle Low | National (80 countries) | Policy and Program: Infant and young child nutrition | Various: broad community, government health facilities | Author generated guidance that draws primarily from experiential knowledge and integrates literature where possible. | Provides guidance to strengthen monitoring systems of infant and young child feeding (IYCF) programs. | Jefferds (2017)^94^ |
| **A programme science-informed framework for HIV prevention** | Monitoring as one component | Lower middle (examples) | Unclear (Implies national /regional) | Program: Sexual, reproductive and HIV health; | Broad community | Expert panel, author generated guidance, drawing from the literature and experiential knowledge. | Describes development of a framework and discusses monitoring dimensions of programme coverage: availability, contact, and utilisation. | McClarity et al. (2023)^95^ |
| **Process evaluation of obesity prevention program in the US (B'More Healthy Communities for Kids)** | guidance /recommendations (indirect) | High | Local (28 neighbourhoods, Baltimore City, USA) | Program: Childhood obesity, nutrition | Various: schools, food stores, restaurants, and households | Author generated features in the methods drawing from the literature. | Routine process evaluation described in methods. | Gittelsohn et al. (2015)^96^ |
| **Scale-up of schools’ behaviour management framework in Maryland USA** | Monitoring as one component | High | State (Maryland, USA) | Program: Mental health | Schools | Author generated description of monitoring in the methods /case study. | Describes the use of monitoring data to optimise implementation of the school-based intervention and school fidelity to the program. | Bradshaw et al. (2014)^97^ |

**References**

1.	Conte KP, Groen S, Loblay V, Green A, Milat A, Persson L, et al. Dynamics behind the scale up of evidence-based obesity prevention: protocol for a multi-site case study of an electronic implementation monitoring system in health promotion practice. Implementation Science. 2017 Dec 6;12(1):146.

2.	Conte KP, Shahid A, Grøn S, Loblay V, Green A, Innes-Hughes C, et al. Capturing implementation knowledge: applying focused ethnography to study how implementers generate and manage knowledge in the scale-up of obesity prevention programs. Implementation Sci. 2019 Dec;14(1):91.

3.	Conte K, Marks L, Loblay V, Grøn S, Green A, Innes-Hughes C, et al. Can an electronic monitoring system capture implementation of health promotion programs? A focussed ethnographic exploration of the story behind program monitoring data. BMC Public Health. 2020 Jun 12;20(1):917.

4.	Goldberg E, Conte K, Loblay V, Groen S, Persson L, Innes-Hughes C, et al. Scale-up of prevention programmes: sustained state-wide use of programme delivery software is explained by normalised self-organised adoption and non-adoption. Implement Sci. 2022 Jan 15;17:5.

5.	Green AM, Innes-Hughes C, Rissel C, Mitchell J, Milat AJ, Williams M, et al. Codesign of the Population Health Information Management System to measure reach and practice change of childhood obesity programs. Public Health Research and Practice [Internet]. 2018 Sep 27 [cited 2024 Jul 19];28(3). Available from: http://www.scopus.com/inward/record.url?scp=85054544333&partnerID=8YFLogxK

6.	Bravo A, Foley BC, Innes-Hughes C, O’Hara BJ, McGill B, Rissel C. The equitable reach of a universal, multisector childhood obesity prevention program (Live Life Well@ School) in Australian primary schools. Public health research & practice. 2020;30(1):e3012003.

7.	Loblay V, Conte KP, Grøn S, Green A, Innes-Hughes C, Persson L, et al. ‘Old’ tools in a new era: unpacking the roles of promotional and informational resources in scaled-up preventive interventions. Critical Public Health. 2022 May 27;32(3):333–44.

8.	Grøn S, Loblay V, Conte KP, Green A, Innes-Hughes C, Milat A, et al. Key performance indicators for program scale-up and divergent practice styles: a study from NSW, Australia. Health Promotion International. 2020;35(6):1415–26.

9.	Farrell L, Lloyd B, Matthews R, Bravo A, Wiggers J, Rissel C. Applying a performance monitoring framework to increase reach and adoption of children’s healthy eating and physical activity programs. Public Health Res Pract. 2014 Nov 28;25(1):e2511408.

10.	Innes-Hughes C, Rissel C, Thomas M, Wolfenden L. Reflections on the NSW healthy children Initiative: A comprehensive state-delivered childhood obesity prevention initiative. Public Health Research and Practice [Internet]. 2019 Mar [cited 2024 Jul 19];29(1). Available from: http://www.scopus.com/inward/record.url?scp=85063126348&partnerID=8YFLogxK

11.	Frieden TR. Six components necessary for effective public health program implementation. Am J Public Health. 2014 Jan;104(1):17–22.

12.	Weaver C, DeRosier ME. Commentary on Scaling-Up Evidence-Based Interventions in Public Systems. Prev Sci. 2019 Nov 1;20(8):1178–88.

13.	World Health Organization. Monitoring flour fortification to maximize health benefits: a manual for millers, regulators, and programme managers [Internet]. World Health Organization; 2021 [cited 2024 Jul 23]. Available from: https://pesquisa.bvsalud.org/portal/resource/pt/who-344903

14.	Pena-Rosas JP, Parvanta I, Van Der Haar F, Chapel TJ. Monitoring and evaluation in flour fortification programs: design and implementation considerations. Nutrition Reviews. 2008;66(3):148–62.

15.	Young AC, Lowry G, Mumford K, Graaf C. CDC’s Prevention Status Reports: Monitoring the Status of Public Health Policies and Practices for Improved Performance and Accountability. Journal of Public Health Management and Practice. 2018 Mar;24(2):121–8.

16.	Fagan AA, Hanson K, Hawkins JD, Arthur MW. Bridging science to practice: Achieving prevention program implementation fidelity in the Community Youth Development Study. American Journal of Community Psychology. 2008;41:235–49.

17.	Fagan AA, Hanson K, Hawkins JD, Arthur MW. Implementing Effective Community-Based Prevention Programs in the Community Youth Development Study. Youth Violence and Juvenile Justice. 2008 Jul;6(3):256–78.

18.	Fagan AA, Bumbarger BK, Barth RP, Bradshaw CP, Cooper BR, Supplee LH, et al. Scaling up Evidence-Based Interventions in US Public Systems to Prevent Behavioral Health Problems: Challenges and Opportunities. Prev Sci. 2019 Nov;20(8):1147–68.

19.	Balvanz P, Singh B, Mwikoko G, Yamanis TJ, Kilonzo MN, Conserve DF, et al. Development and implementation of a process evaluation for intervention quality improvement of a community-based behavioral HIV intervention trial in Tanzania. J Public Health (Berl). 2023 Jun;31(6):877–84.

20.	Barua A, Watson K, Plesons M, Chandra-Mouli V, Sharma K. Adolescent health programming in India: a rapid review. Reprod Health. 2020 Dec;17(1):87.

21.	Bhattacharjee P, Musyoki HK, Becker M, Musimbi J, Kaosa S, Kioko J, et al. HIV prevention programme cascades: insights from HIV programme monitoring for female sex workers in Kenya. J Intern AIDS Soc. 2019 Jul;22(S4):e25311.

22.	Brinsden H, Lobstein T, Landon J, Kraak V, Sacks G, Kumanyika S, et al. Monitoring policy and actions on food environments: rationale and outline of the INFORMAS policy engagement and communication strategies. Obesity Reviews. 2013 Oct;14(S1):13–23.

23.	Mackay S, Gerritsen S, Sing F, Vandevijvere S, Swinburn B. Implementing healthy food environment policies in New Zealand: nine years of inaction. Health Res Policy Sys. 2022 Dec;20(1):8.

24.	Swinburn B, Vandevijvere S, Kraak V, Sacks G, Snowdon W, Hawkes C, et al. Monitoring and benchmarking government policies and actions to improve the healthiness of food environments: a proposed G overnment H ealthy F ood E nvironment P olicy I ndex. Obesity Reviews. 2013 Oct;14(S1):24–37.

25.	Vandevijvere S, Mackay S, Swinburn B. Measuring and stimulating progress on implementing widely recommended food environment policies: the New Zealand case study. Health Res Policy Sys. 2018 Dec;16(1):3.

26.	Yamaguchi M, Nomura M, Arai Y, Vandevijvere S, Swinburn B, Nishi N. An assessment of implementation gaps and priority recommendations on food environment policies: the Healthy Food Environment Policy Index in Japan. Public Health Nutrition. 2022;25(6):1720–32.

27.	Swinburn B, Sacks G, Vandevijvere S, Kumanyika S, Lobstein T, Neal B, et al. INFORMAS (International Network for Food and Obesity/non communicable diseases Research, Monitoring and Action Support): overview and key principles. Obesity Reviews. 2013 Oct;14(S1):1–12.

28.	Boileau-Falardeau M, Farooqi S, O’Rourke C, Payne L. Revisiting the Innovation Strategy performance measurement process: insights from practice. Canadian Journal of Public Health. 2021;112(Suppl 2):262–9.

29.	DeGroff A, Schooley M, Chapel T, Poister TH. Challenges and strategies in applying performance measurement to federal public health programs. Evaluation and Program Planning. 2010;33(4):365–72.

30.	DeGroff AS. New public management and governance collide: federal-level performance measurement in networked public management environments [Internet]. Georgia Institute of Technology and Georgia State University; 2009 [cited 2024 Jul 23]. Available from: https://search.proquest.com/openview/e461805bb77f11a8461dafc82eff2db3/1?pq-origsite=gscholar&cbl=18750&casa_token=m2qIQH_IZR4AAAAA:JWxHooGGlmshPoB9XcvJENieJB0k_72_5TBUX3_-kuyklGMT_UIz7aFIMPb011ROngSkZ7o9iCHl

31.	Bryant CA, Courtney AH, McDermott RJ, Alfonso ML, Baldwin JA, Nickelson J, et al. Promoting Physical Activity Among Youth Through Community-Based Prevention Marketing. Journal of School Health. 2010 May;80(5):214–24.

32.	Bryce J, Roungou JB, Nguyen-Dinh P, Naimoli JF, Breman JG. Evaluation of national malaria control programmes in Africa. Bulletin of the World Health Organization. 1994;72(3):371.

33.	Cole DC, Levin C, Loechl C, Thiele G, Grant F, Girard AW, et al. Planning an integrated agriculture and health program and designing its evaluation: experience from Western Kenya. Evaluation and Program Planning. 2016;56:11–22.

34.	DeRosier ME. Three critical elements for real-time monitoring of implementation and adaptation of prevention programs. The journal of primary prevention. 2019;40:129–35.

35.	Funk M, Freeman M. Framework and methodology for evaluating mental health policy and plans. Health Planning & Management. 2011 Apr;26(2):134–57.

36.	Hussain B, Hui A, Timmons S, Nkhoma K. Ethnic mental health inequalities and mental health policies in England 1999-2020. Journal of Public Mental Health. 2022;21(2):162–73.

37.	Glassman M, Lacson R, Collins B, Hill C, Wan C. Lessons Learned from the First Year of Implementation of the Centers for Disease Control and Prevention’s Standardized Evaluation System for HIV Prevention Programs. AIDS Education and Prevention. 2002 Jun;14(3_supplement):49–58.

38.	Jain N, Bahl D, Mehta R, Bassi S, Sharma K, Arora M. Progress and challenges in implementing adolescent and school health programmes in India: a rapid review. BMJ open. 2022;12(5):e047435.

39.	Lakerveld J, Woods C, Hebestreit A, Brenner H, Flechtner-Mors M, Harrington JM, et al. Advancing the evidence base for public policies impacting on dietary behaviour, physical activity and sedentary behaviour in Europe: the Policy Evaluation Network promoting a multidisciplinary approach. Food Policy. 2020;96:101873.

40.	Lee CYS, August GJ, Realmuto GM, Horowitz JL, Bloomquist ML, Klimes-Dougan B. Fidelity at a distance: Assessing implementation fidelity of the Early Risers Prevention Program in a going-to-scale intervention trial. Prevention Science. 2008;9:215–29.

41.	Le Loup G, Fleury S, Camargo K, Larouzé B. International institutions, Global Health Initiatives and the challenge of sustainability: lessons from the Brazilian AIDS programme. Tropical Medicine & International Health [Internet]. 2009 Oct [cited 2024 Jul 22]; Available from: https://onlinelibrary.wiley.com/doi/10.1111/j.1365-3156.2009.02411.x

42.	Merrill JC, Pinsky I, Killeya-Jones LA, Sloboda Z, Dilascio T. Substance abuse prevention infrastructure: a survey-based study of the organizational structure and function of the D.A.R.E. program. Subst Abuse Treat Prev Policy. 2006 Dec;1(1):25.

43.	McGraw SA, Sellers D, Stone E, Resnicow KA, Kuester S, Fridinger F, et al. Measuring implementation of school programs and policies to promote healthy eating and physical activity among youth. Preventive Medicine. 2000;31(2):S86–97.

44.	Palinkas LA, Spear SE, Mendon SJ, Villamar J, Valente T, Chou CP, et al. Measuring sustainment of prevention programs and initiatives: a study protocol. Implementation Sci. 2015 Dec;11(1):95.

45.	Rickrode-Fernandez Z, Kao J, Lesser MN, Guess K. Implementation of a healthy food and beverage policy at a public university. Journal of Nutrition Education and Behavior. 2021;53(10):891–9.

46.	Rotheram-Borus MJ, Swendeman D, Chorpita BF. Disruptive innovations for designing and diffusing evidence-based interventions. American Psychologist. 2012;67(6):463.

47.	Salmela R. Health policies and health for all strategies in the Nordic countries. Health policy. 1991;18(3):207–18.

48.	Schultz L, Ruel-Bergeron J. Considerations for monitoring school health and nutrition programs. Frontiers in public health. 2021;9:645711.

49.	Sgaier SK, Anthony J, Bhattacharjee P, Baer J, Malve V, Bhalla A, et al. Strengthening government management capacity to scale up HIV prevention programs through the use of Technical Support Units: lessons from Karnataka state, India. Global Health: Science and Practice. 2014;2(4):444–58.

50.	Thomas CW, Smith BD, Wright-DeAgüero L. The Program Evaluation and Monitoring System: A Key Source of Data for Monitoring Evidence–Based HIV Prevention Program Processes and Outcomes. AIDS Education and Prevention. 2006 Aug;18(supp):74–80.

51.	Turner C, Yorkston E, Hart K, Drew L, McClure R. Simplifying data collection for process evaluation of community coalition activities – an electronic web-based application. Health Promot J Aust. 2006;17(1):48–53.

52.	WHO. Monitoring, evaluation and review of national health strategies: a country-led platform for information and accountability. 2011.

53.	Zakarija-Grković I, Cattaneo A, Bettinelli ME, Pilato C, Vassallo C, Borg Buontempo M, et al. Are our babies off to a healthy start? The state of implementation of the Global strategy for infant and young child feeding in Europe. Int Breastfeed J. 2020 Dec;15(1):51.

54.	Danforth K, Ahmad AM, Blanchet K, Khalid M, Means AR, Memirie ST, et al. Monitoring and evaluating the implementation of essential packages of health services. BMJ global health. 2023;8(Suppl 1):e010726.

55.	Fernald D, Harris A, Deaton EA, Weister V, Pray S, Baumann C, et al. Peer reviewed: a standardized reporting system for assessment of diverse public health programs. Preventing Chronic Disease [Internet]. 2012 [cited 2024 Jul 22];9. Available from: https://www.ncbi.nlm.nih.gov/pmc/articles/PMC3475516/

56.	L’Engle K, Plourde KF, Zan T. Evidence-based adaptation and scale-up of a mobile phone health information service. Mhealth [Internet]. 2017 [cited 2024 Jul 22];3. Available from: https://www.ncbi.nlm.nih.gov/pmc/articles/PMC5427190/

57.	Meghani AN. Examining the Policy and Practice Disconnect: The Impact of Data-Driven Performance Monitoring on Data Quality in India’s Public Health Sector [Internet] [PhD Thesis]. The Johns Hopkins University; 2020 [cited 2024 Jul 22]. Available from: https://search.proquest.com/openview/2bcd15294c8b7856197b4d05c35fd625/1?pq-origsite=gscholar&cbl=18750&diss=y&casa_token=a6xlHKZrZRUAAAAA:CB25G_qlRIzwUg-RQWxjZ3Jhz9Yhi2TT0JujApfBYLma78-BUva4aJ9utFCoEQcmRK_O0MWUgXek

58.	Sacks E, Swanson RC, Schensul JJ, Gleave A, Shelley KD, Were MK, et al. Community Involvement in Health Systems Strengthening to Improve Global Health Outcomes: A Review of Guidelines and Potential Roles. Int Q Community Health Educ. 2017 Jul;37(3–4):139–49.

59.	Saunders RP, Evans MH, Joshi P. Developing a Process-Evaluation Plan for Assessing Health Promotion Program Implementation: A How-To Guide. Health Promotion Practice. 2005 Apr;6(2):134–47.

60.	Wiessing L, Ferri M, Běláčková V, Carrieri P, Friedman SR, Folch C, et al. Monitoring quality and coverage of harm reduction services for people who use drugs: a consensus study. Harm Reduct J. 2017 Dec;14(1):19.

61.	WHO,. Communicable disease surveillance and response systems: Guide to monitoring and evaluating. 2006. Report No.: WHO/CDS/EPR/LYO/2006.2.

62.	World Health Organization. Netcode toolkit: monitoring the marketing of breast-milk substitutes: protocol for ongoing monitoring systems [Internet]. World Health Organization; 2017 [cited 2024 Jul 23]. Available from: https://apps.who.int/iris/bitstream/handle/10665/259441/9789241513180-eng.pdf

63.	International Health Regulations. IHR MONITORING AND EVALUATION FRAMEWORK: Geneva: World Health Organization; 2018. Licence: CC BY-NC-SA 3.0 IGO. 2005.

64.	World Health Organization. Monitoring and evaluating digital health interventions: a practical guide to conducting research and assessment [Internet]. World Health Organization; 2016 [cited 2024 Jul 23]. Available from: https://apps.who.int/iris/bitstream/handle/10665/252183/?sequence=1

65.	World Health Organization. Indicator and Monitoring Framework for the Global Strategy for Women’s, Children’s and Adolescent’s Health (2016–2030). In: Every Woman Every Child. 2016.

66.	Home Fortification Technical Advisory Group. A manual for developing and implementing monitoring systems for home fortification interventions. Geneva: Home Fortification Technical Advisory Group; 2013.

67.	Centers for Disease Control and Prevention. Results from the school health policies and practices study. 2016.

68.	CDC Nutrition,. CDC Nutrition, Monitor and Evaluate [Internet]. CDC Nutrition, Monitor and Evaluate. 2024. Available from: https://www.cdc.gov/nutrition/food-service-guidelines/monitor-and-evaluate/index.html

69.	Global Alliance for Improved Nutrition (GAIN), Project Healthy Children (PHC), Iodine Global Network (IGN), Food Fortification Initiative (FFI), Technoserve. Regulatory monitoring of national food fortification programs: A policy guidance document. UNICEF; 2018.

70.	UNICEF, GAIN. Brighter Futures: Protecting early brain development through salt iodization-The UNICEF-GAIN Partnership Project. 2018.

71.	Aqil A, Silvestre E, Hotchkiss D, Maniscalco L. Health Systems Strengthening–Monitoring, Evaluation, and Learning Guide. 2017.

72.	USAID. ADS Chapter 201 Program Cycle Operational Policy [Internet]. < bound method Organization. get_name_with_acronym of< Organization: United …; 2022 [cited 2024 Jul 23]. Available from: https://policycommons.net/artifacts/4307324/ads-chapter-201-program-cycle-operational-policy/5117695/

73.	Centre for Epidemiology and Evidence. Increasing the Scale of Population Health Interventions: A Guide. NSW Ministry of Health; 2014.

74.	Conte KP, Hawe P. Will E-Monitoring of Policy and Program Implementation Stifle or Enhance Practice? How Would We Know? Front Public Health. 2018 Sep 11;6:243.

75.	Flora JA, Lefebvre RC, Murray DM, Stone EJ, Assaf A, Mittelmark MB, et al. A community education monitoring system: methods from the Stanford Five-City Project, the Minnesota Heart Health Program and the Pawtucket Heart Health Program. Health Education Research. 1993;8(1):81–95.

76.	Mpofu M, Semo B werq, Grignon J, Lebelonyane R, Ludick S, Matshediso E, et al. Strengthening monitoring and evaluation (M&E) and building sustainable health information systems in resource limited countries: lessons learned from an M&E task-shifting initiative in Botswana. BMC Public Health. 2014 Dec;14(1):1032.

77.	Samuels T, Kirton J, Guebert J. Monitoring compliance with high-level commitments in health: the case of the CARICOM Summit on Chronic Non-Communicable Diseases. Bulletin of the World Health Organization. 2014;92:270-276B.

78.	Vaughan M, Jernigan J, Barnes SP, Shea P, Davis R, Rutledge S. Evaluating cross-cutting approaches to chronic disease prevention and management: developing a comprehensive evaluation. Preventing Chronic Disease. 2017;14:E131.

79.	Yousafzai AK, Rasheed MA, Siyal S. Integration of parenting and nutrition interventions in a community health program in Pakistan: an implementation evaluation. Annals of the New York Academy of Sciences. 2018 May;1419(1):160–78.

80.	UNAIDS. 12 Components Monitoring & Evaluation System Assessment. In Geneva; 2009 [cited 2024 Jul 23]. (Guidelines to Support Preparation, Implementation and Follow-Up Activities). Available from: https://www.google.com/search?q=12+Components+Monitoring+%26+Evaluation+System+Assessment+Guidelines+to+Support+Preparation%2C+Implementation+and+Follow-Up+Activities+These+guidelines+support+the+use+of+the+standardized+tool%3A+12+Components+Monitoring+and+Evaluation+System+Strengthening+Tool.+Geneva%3A+UNAIDS%2C+2009a&rlz=1C1VDKB_en-gbAU1103AU1103&oq=12+Components+Monitoring+%26+Evaluation+System+Assessment+Guidelines+to+Support+Preparation%2C+Implementation+and+Follow-Up+Activities+These+guidelines+support+the+use+of+the+standardized+tool%3A+12+Components+Monitoring+and+Evaluation+System+Strengthening+Tool.+Geneva%3A+UNAIDS%2C+2009a&gs_lcrp=EgZjaHJvbWUyBggAEEUYOTIGCAEQRRg80gEHMzY2ajBqN6gCALACAA&sourceid=chrome&ie=UTF-8

81.	Hardy LL, King L, Espinel P, Cosgrove C, Bauman A. NSW Schools Physical Activity and Nutrition Survey (SPANS) 2010: Full Report. [Internet]. Sydney: NSW Ministry of Health; 2010. Available from: www.health.nsw.gov.au or www.sydney.edu.au/medicine/public-health/panorg/

82.	UNESCO,. Monitoring and evalution guidance for school halth programs: Eight Core Indicators to Support FRESH (Focusing Resources on Effective School Health) [Internet]. 2014. Available from: www.unesco.org/new/health-education

83.	World Health Organization. WHO report on the global tobacco epidemic 2017: Monitoring tobacco use and prevention policies [Internet]. World Health Organization; 2017 [cited 2024 Jul 23]. Available from: https://escholarship.org/content/qt8nw5p0zt/qt8nw5p0zt.pdf

84.	Whiting S, Mendes R, Morais ST, Gelius P, Abu-Omar K, Nash L, et al. Promoting health-enhancing physical activity in Europe: surveillance, policy development and implementation 2015–2018. Health Policy. 2021;125(8):1023–30.

85.	Win Tin ST, Kubuabola I, Snowdon W, Cash HL, Na’ati E, Waqa GD, et al. Assessing the progress on the implementation of policy and legislation actions to address the Non-Communicable Diseases crisis in the Pacific. Mockridge J, editor. PLoS ONE. 2022 Aug 11;17(8):e0272424.

86.	Tolley H, Snowdon W, Wate J, Durand AM, Vivili P, McCool J, et al. Monitoring and accountability for the Pacific response to the non-communicable diseases crisis. BMC Public Health. 2016 Dec;16(1):958.

87.	Win Tin ST, Kubuabola I, Ravuvu A, Snowdon W, Durand AM, Vivili P, et al. Baseline status of policy and legislation actions to address non communicable diseases crisis in the Pacific. BMC Public Health. 2020 Dec;20(1):660.

88.	Reynolds HW, Sutherland EG. A systematic approach to the planning, implementation, monitoring, and evaluation of integrated health services. BMC Health Serv Res. 2013 Dec;13(1):168.

89.	Lane J, Verani A, Hijazi M, Hurley E, Hagopian A, Judice N, et al. Monitoring HIV and AIDS related policy reforms: a road map to strengthen policy monitoring and implementation in pepfar partner countries. PLoS One. 2016;11(2):e0146720.

90.	Biradavolu MR, Blankenship KM, George A, Dhungana N. Unintended consequences of community-based monitoring systems: Lessons from an HIV prevention intervention for sex workers in South India. World Development. 2015;67:1–10.

91.	Levinson FJ, Madzorera I. Recent experience in the monitoring and evaluation of nutrition-related projects in developing countries: nine lessons learned. Food policy and applied nutrition program discussion paper [Internet]. 2005 [cited 2024 Jul 22];(28). Available from: https://nutrition.tufts.edu/sites/default/files/fpan/wp28-mep_nine_lessons.pdf

92.	Ruel-Bergeron JC, Hurley KM, Kang Y, Aburto N, Farhikhtah A, Dinucci A, et al. Monitoring and evaluation design of Malawi’s Right Foods at the Right Time nutrition program. Evaluation and program planning. 2019;73:1–9.

93.	Volf K, Kelly L, Van Hoye A, García Bengoechea E, MacPhail A, Woods CB, et al. Assessing the implementation of physical activity-promoting public policies in the Republic of Ireland: a study using the Physical Activity Environment Policy Index (PA-EPI). Health Res Policy Sys. 2023 Jun 26;21(1):63.

94.	Jefferds MED. Government information systems to monitor complementary feeding programs for young children. Maternal & Child Nutrition. 2017 Oct;13(S2):e12413.

95.	McClarty LM, Becker ML, García PJ, Garnett GP, Dallabetta GA, Ward H, et al. Programme science: a route to effective coverage and population-level impact for HIV and sexually transmitted infection prevention. The Lancet HIV. 2023 Dec;10(12):e825–34.

96.	Gittelsohn J, Yang T, Kharmats A, Steeves EA, Rastatter S, Welsh C, et al. Process Evaluation to Monitor and Guide a Multi‐Level, Multi‐Component Obesity Prevention Program for Low‐Income, African American Youth. The FASEB Journal. 2015 Apr;29(S1):584.22.

97.	Bradshaw C, J Debnam K, Lindstrom Johnson S, T Pas E, Hershfeldt P, Alexander A, et al. Maryland’s evolving system of social, emotional, and behavioral interventions in public schools: The Maryland Safe and Supportive Schools Project. Adolescent Psychiatry. 2014;4(3):194–206.
